# Investigation of Red Blood Cell Properties on Impedance Signatures Generated in a Coulter Counter

**DOI:** 10.1002/cnm.70172

**Published:** 2026-05-07

**Authors:** Pierre Pottier, Pierre Taraconat, Jean‐Philippe Gineys, Damien Isèbe, Franck Nicoud, Simon Mendez

**Affiliations:** ^1^ Horiba ABX SAS Grabels France; ^2^ Institut Montpellierain Alexander Grothendieck CNRS, University of Montpellier Montpellier France; ^3^ Institut Universitaire de France (IUF) Paris France

**Keywords:** computational fluid dynamics, Coulter counter, mesh adaptation, red blood cell

## Abstract

The ability of red blood cells (RBCs) to deform is essential for microcirculation and oxygen delivery. Any changes in RBC deformability can lead to significant cardiovascular complications, necessitating timely detection. Although specialized microdevices can be designed to assess RBC deformability, leveraging instruments already used in clinical settings would enable easier integration and accelerate clinical translation. Coulter counter (CC) systems are routinely used to count, size, and analyze RBCs and the possibility to extend their diagnostic capabilities to RBC deformability is currently examined. In this study, the effects of RBCs' geometrical, morphological, and rheological properties on CC measurement have been investigated numerically, thanks to a simulation framework predicting the RBC dynamics in a CC and the associated impedance signature. Subsequently, a numerical parametric study has been performed, and the resulting pulses have been compared with experimental results, confirming the simulation's accuracy in predicting CC measurements. In addition to the RBC volume and the RBC trajectory in the sensing region, which had been investigated before, present results show that in our modeling framework, RBC sphericity, membrane viscosity, and cytoplasm viscosity are the main RBC characteristics contributing to the broad CC measurement spectrum observed experimentally when analyzing healthy blood samples.

## Graphical abstract

Numerical simulations predicting red blood cell (RBC) dynamics and their electrical signatures in a Coulter counter have been performed, enhancing our understanding of how RBC deformability influences these measurements—traditionally used for counting and sizing. Supported by experimental results, the numerical study conducted confirms the role of RBC morpho‐rheological properties on signatures shape and helps us identify the main contributors to signature shape variability in healthy RBCs, namely: RBC sphericity, cytoplasm viscosity, and membrane viscosity.

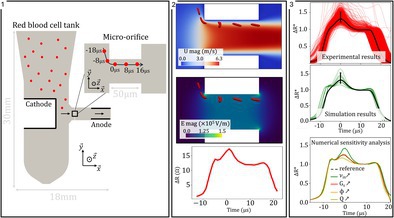

**Abbreviations**
CBC: complete blood count
CC: Coulter counter
FSI: fluid–structure interaction
IBM: immersed boundary method
MA: mesh adaptation RBC, red blood cell

## 1 Introduction

Healthy red blood cells (RBCs) have the property to significantly change their shape under external stresses. Their high deformability plays a key role in blood circulation and oxygen delivery, especially in microcirculation where RBCs circulate through capillaries narrower than their size at rest [1]. Moreover, severe cardiovascular dysfunction can occur when the deformability of RBCs is impaired. For example, anemias, organ ischemia or hypertension may develop in patients with pathologies affecting RBC deformability such as spherocytosis [2, 3], sickle cell disease [4, 5] or diabetes mellitus [1, 6]. The detection of abnormal RBC deformability is thus crucial for predicting and preventing major cardiovascular complications. Among the methods used for measuring RBC deformability at the single cell level, the manual micropipette [7, 8], optical tweezers [9, 10] and atomic force microscopy [11, 12] are among the most widely used. High‐throughput automated techniques are also available, such as ektacytometry [3, 13] and microfluidic systems [14, 15]. To the best of our knowledge, none of these methods are implemented in routine analysis, although they can be employed for specific diagnostic purposes [16, 17].

**FIGURE 1:**
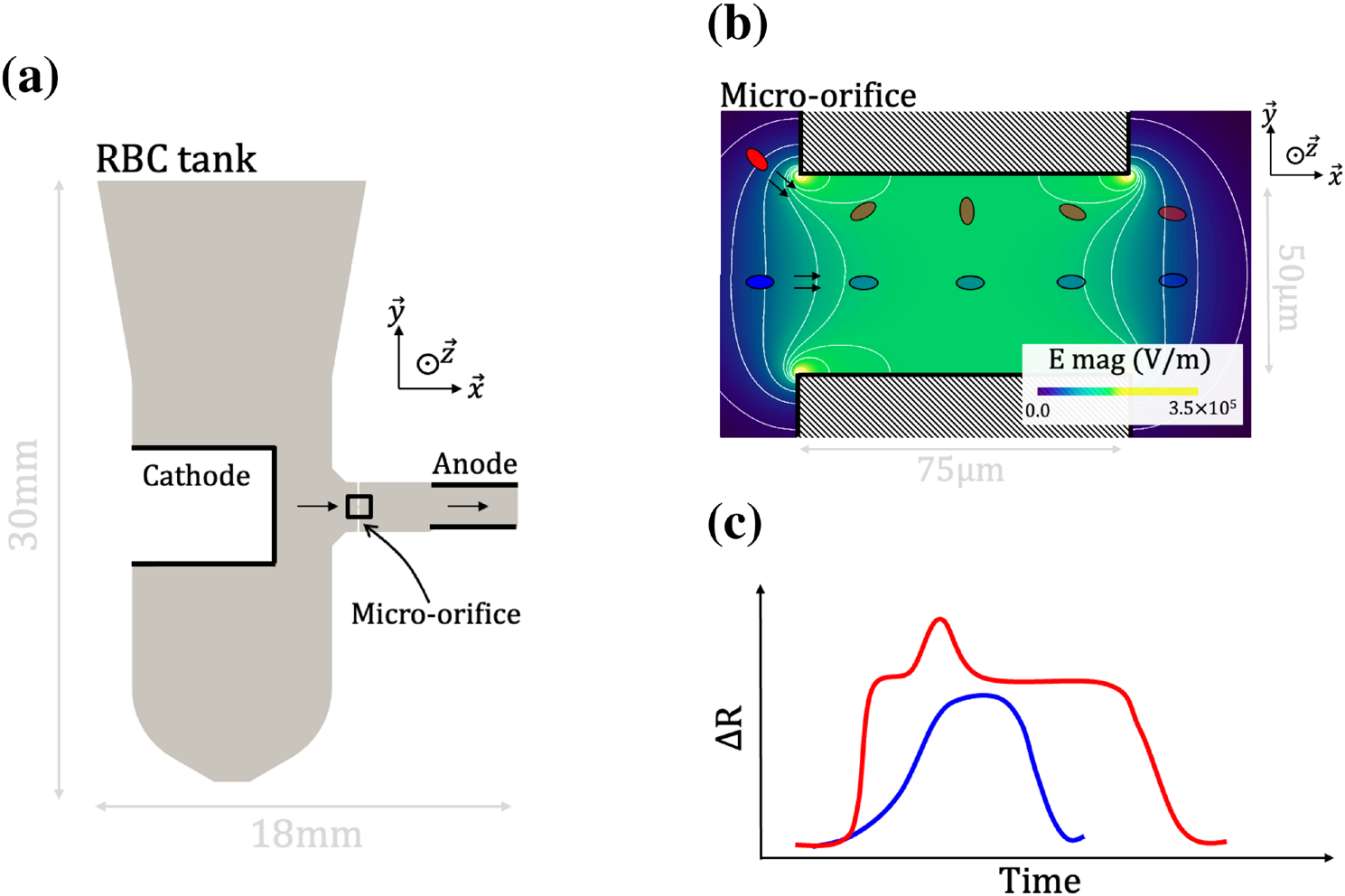
Coulter principle illustrated with a schematic version of the ABX Micros 60 (HORIBA) analyzer. (a) Slice cut of RBC tank. The electrodes are highlighted with black solid lines, positioned on each side of a rectangle that indicates the location of the cylindrical orifice. (b) Slice cut of the micro‐orifice. The electric field magnitude is mapped on the micro‐orifice picture (cell‐free). Electric field isolines are shown with white lines. The blue and red dots illustrate the time‐lapse positions of two different RBC moving through the orifice, with different trajectories. (c) Time evolution of the electric perturbations associated with the passage of RBCs through the micro‐orifice. The blue pulse represents the electric perturbation caused by the blue particle (central path), while the red pulse corresponds to the red particle (near‐wall path).

A routine and widely used blood test is the complete blood count (CBC), which includes RBC count and size. Figure 1a–c illustrate the principle of an automated electric impedance‐based system, the Coulter counter (CC) [18], commonly used for this purpose. In a CC, RBCs are diluted and suspended in an electrolyte reagent in a tank presented in Figure 1a, then drawn through a micro‐orifice shown in Figure 1b. Electrodes on either side of the micro‐orifice generate an electric field in the system. When the RBCs flow through the orifice, they cause a variation in the electric resistance of the system, referred to as “pulse”. This mechanism is illustrated in Figure 1b, showing two particles (in different colors) following distinct trajectories. The corresponding electric perturbations are presented in Figure 1c, with colors consistent with those of the associated particles. To ensure high throughput, RBCs travel through the orifice at speeds of several meters per second, with pulse durations on the order of tens of microseconds [19].

Consistent with experimental observations, the following relation has been proposed by Kachel et al. [20] to assess a resistive pulse ΔR for an insulating particle flowing in a CC:

(1) $$\Delta R\propto f_{s}V,$$

where V is the volume of the particle and $f_{s}$ a shape factor that accounts for the particle size, orientation and shape. Focusing on hydrodynamical effects, it has been shown that non‐spherical particles exhibit different dynamics depending on their proximity to the wall, resulting in varying pulse shapes for the same particle [19, 21]. Particles flowing in the central region (blue particle in Figure 1b) remain aligned with the flow direction and generate standard bell‐shaped pulses (blue pulse in Figure 1c), whose maximum amplitude is used as a measurement of cell volume [20]. In contrast, particles flowing near the orifice wall (red particle in Figure 1b) are subjected to strong shear forces, causing them to rotate and deform, which yields an additional peak occurring during cell rotation (red pulse in Figure 1c). This “rotation peak”, which increases the pulse's maximum amplitude, is induced by an increase of $f_{s}$ [19, 20]. In systems where these types of artifacts occur, the measured volume distribution is abnormally skewed to the right and fails to accurately represent the true statistics of RBCs volume [22]. Various strategies have been proposed to correct volume distribution, including hydrofocused systems, that force RBCs to pass through the central region of the orifice [23], or pulse editing strategies, designed to filter out near‐wall pulses [22, 24].

Recent studies demonstrated the sensitivity of pulses with a rotation peak to RBC deformability: Taraconat et al. [25] showed that the rotation peak increases if the RBC is rigidified, or is practically nonexistent if the RBC is spherized. These results have been confirmed experimentally with analyses conducted on artificially modified RBCs [25]. In addition, the pulses with rotation peak have been used to discriminate healthy blood samples from pathological blood samples containing RBCs with altered deformability [6].

In the long term, the objective is to enhance CC diagnostic capabilities by identifying morphological and rheological RBC properties from the CC measurements. However, the relationship between the RBCs characteristics and the impedance signatures has not been fully elucidated. Indeed, the numerical study conducted by Taraconat et al. [25] considered a simplified model for the RBC, in which RBC membrane viscosity was neglected. However, RBC membrane viscoelasticity is expected to play an important role in such configurations, where RBCs are subjected to rapid variations of stress. The role of RBC initial orientation in the RBC tank (before the vacuuming phase) was not discussed either. Of course, interpreting the impedance signatures in terms of RBC characteristics and dynamics requires: (1) identifying all factors responsible for variations in pulse shape, and (2) ensuring that numerical simulations can reproduce the pulse variations observed experimentally.

This study focuses on the two prerequisites outlined above. To do so, we propose a numerical approach to explore how the geometrical, morphological, and rheological properties of RBCs, including the viscosity of the RBC membrane and their initial orientation, influence resistance pulses. The numerical framework is based on the work of Taraconat et al. [19], enhanced with a mesh adaptation (MA) strategy for improving computation efficiency. First, the numerical framework and the experimental set up are presented in Section 2. Following the numerical model's validation in Section 3.1, the influence of RBCs properties is investigated through a one‐at‐a‐time sensitivity analysis at multiple operating points in Section 3.2. Subsequently, a numerical systematic parametric study has been performed with all RBC properties identified, and the results are compared with experimental data in Section 3.3. Finally, the model's ability to capture the experimental pulses are discussed in Section 4.

## 2 Materials and Methods

### 2.1 Numerical Simulations

Simulations are performed with YALES2BIO (http://imag.umontpellier.fr/~yales2bio), a numerical simulation software dedicated to blood flow simulations at different scales [26]. Predicting impedance pulses in a CC first necessitates solving a fluid–structure interaction (FSI) problem involving the RBC and the ambient fluid. Then, the resistive pulses associated with the RBC dynamics can be computed. In a real device, the blood samples are sufficiently diluted so that RBCs flow in the micro‐orifice one after the other. Coincident passages can easily be filtered. We thus focus on the case of an isolated RBC with variable properties flowing in a CC. The model and the numerical method for both the FSI and the electric problems are summarized in Section 2.1.1. A mesh adaptation strategy is proposed to reduce the computational cost of the simulation in Section 2.1.2. Section 2.1.3 summarizes the pipeline.

#### 2.1.1 Modeling Framework

##### 2.1.1.1 Fluid–Structure Interaction Calculation

Predicting the dynamics of an RBC in a CC requires solving a fluid–structure interaction problem between the incompressible suspending electrolyte and the RBC. The latter is modeled as a droplet of Newtonian fluid (the cytoplasm) enclosed by an infinitely thin, deformable solid structure representing the membrane. The volume of the RBC $V_{\mathrm{RBC}}$ is fixed (the membrane is assumed impermeable) and the surface at rest is denoted by $S_{\mathrm{RBC}}$. As the lipid bilayer is quasi‐incompressible, $S_{\mathrm{RBC}}$ also remains constant. This allows to define Q, the sphericity index, as: $Q=\frac{V_{\mathrm{RBC}}}{V_{\text{sphere}}}$, with $V_{\text{sphere}}$ the volume of a sphere having the same surface area as the RBC, $S_{\mathrm{RBC}}$. For a given surface, a larger RBC volume results in a more spherical cell, with Q approaching 1.0. On the other hand, Q tends to 0 when the RBC is emptied of its volume and adopts a flattened shape.

From a mechanical point of view, the RBC membrane resists different types of stresses, in particular shear and area changes, modeled here with Skalak's hyperelastic law [27]:

(2) $$W_{\mathrm{SK}}=\frac{G_{s}}{4}\left[\left(\lambda_{1}^{2}+\lambda_{2}^{2}-2\right)+2\left(\lambda_{1}^{2}+\lambda_{2}^{2}-\lambda_{1}^{2}\lambda_{2}^{2}-1\right)\right]+\frac{E_{a}}{4}\left(\lambda_{1}^{2}\lambda_{2}^{2}-1\right).$$

$W_{\mathrm{SK}}$ is the strain energy of the membrane. It is a function of the principal in‐plane strain values ($\lambda_{1}$ and $\lambda_{2}$), the shear modulus $G_{s}$ and the area‐change modulus $E_{a}$. The bending resistance of the membrane is described by the Helfrich bending energy $\varepsilon_{b}$ [28]:

(3) $$\varepsilon_{b}=\frac{E_{b}}{2}\int_{S}{\left(2\kappa -C_{0}\right)}^{2}\mathrm{dS}.$$

$E_{b}$ the bending modulus, κ the mean curvature and $C_{0}$ the spontaneous curvature.

In addition, the RBC membrane has been shown to have a viscoelastic behavior [29, 30]. In YALES2BIO, membrane viscosity is considered through Skalak's law [31], in which the classical shear modulus $G_{s}$ of Equation (2) is replaced by a relaxation modulus $E_{r}\left(t\right)$:

(4) $$E_{r}\left(t\right)=G_{s}+E_{e}e^{-\frac{t}{t_{c}}},$$

where $t_{c}$ refers to a characteristic time, computed as: $t_{c}=\frac{\eta_{\text{memb}}}{E_{e}}$, with $\eta_{\text{memb}}$ the membrane viscosity. In practice, $E_{e}$ is set to $E_{e}=50G_{s}$ [31] to have negligible differences with a Kelvin‐Voigt model, which is the classical model used to characterize membrane viscosity [14, 29]. However, the implementation through a Zener model (Equation 4) is preferred here for stability reasons [31, 32]. As generally assumed in simulations of RBC dynamics, membrane viscosity only resists shear stresses (not area changes). The relaxation modulus $E_{r}\left(t\right)$ is thus included in Skalak's law solely for computing the deviatoric part of the stress tensor [31]. The model does not account for potential stress‐induced RBC damage [33, 34]; therefore, RBC properties remain constant throughout each simulation.

The fluid is assumed to be incompressible and Newtonian. The flow is thus governed by the incompressible Navier–Stokes equations, solved on an Eulerian mesh consisting of tetrahedral elements. The RBC membrane is modeled using a two‐dimensional Lagrangian mesh composed of triangular elements. FSI calculations are carried out using the immersed boundary method (IBM) originally developed by Peskin et al. [35], extended to unstructured grids using the Reproducing Kernel Particle Method (RKPM) [26, 36]. At each time step, RBC membrane forces are calculated from the membrane deformation and the behavior laws (Equations (2), (3), (4)) [31, 37, 38]. Then, membrane forces are regularized over the fluid domain using the RKPM [26]. In addition, the viscosity field is recalculated at each time step at the fluid grid vertices, based on the position of the membrane, in order to assign the cytoplasm viscosity inside the membrane and the electrolyte viscosity outside. The Navier–Stokes equations are thus written as follows:

(5) $$\frac{\partial \vec{u}}{\partial t}+\vec{u}\cdot \nabla \vec{u}=-\frac{\nabla P}{\rho }+\nabla \cdot \left[\nu \left(\nabla \vec{u}+{\left(\nabla \vec{u}\right)}^{t}\right)\right]+\vec{f},$$

(6) $$\nabla \cdot \vec{u}=0,$$

with $\vec{u}$ the fluid velocity, ν the fluid kinematic viscosity (which varies in space), ρ the fluid density and P the pressure. $\vec{f}$ is a source term that represents the effect of RBC membrane forces on the fluid. In the rest of this paper, the kinematic viscosity is denoted by $\nu_{\text{in}}$ inside the RBC and $\nu_{\mathrm{ext}}$ in the suspending medium. Equation (5) is discretized with a fourth‐order finite‐volume method, and advanced in time with a fourth‐order Runge–Kutta scheme. The resolution of the velocity field is achieved thanks to the prediction–correction method from Chorin et al. [39] predicting a velocity field with the previous pressure term, then correcting it based on the pressure Poisson equation (enforcing incompressibility condition). The pressure Poisson equation is solved using a Deflated Preconditioned Conjugate Gradient [40]. Once the velocity field has been calculated on the fluid grid, it is interpolated onto the vertices of the membrane mesh and the RBC membrane is advanced in time with an explicit Euler scheme.

The FSI framework is used to predict the dynamics of an RBC circulating through a CC and needs to be completed by an electric calculation to obtain the associated pulse.

##### 2.1.1.2 Electrostatic Calculation

The CC measurement consists in the time‐dependent variation in the electric resistance of the system during the passage. A separate set of computations is therefore carried out to evaluate the resistance change, $\Delta R\left(t\right)$, associated with an RBC flowing through the micro‐aperture: $\Delta R\left(t\right)=R_{\mathrm{RBC}}\left(t\right)-R_{\mathrm{CF}}.$ $R_{\mathrm{CF}}$ is the resistance computed in a cell‐free configuration, and $R_{\mathrm{RBC}}\left(t\right)$ is the resistance of the system perturbed by an RBC, at time t. The RBC is assumed to be perfectly insulating, which is mimicked in the IBM framework by setting the conductivity inside the RBC $\sigma_{\text{in}}$ to 0. Furthermore, the effects of the electric field on RBCs dynamics are neglected and it is assumed that the electric field develops instantaneously. In this respect, electric effects are decoupled from FSI problem and disturbances caused by an RBC over time may be seen as a series of electrostatic problems to be solved. For each RBC state (defined by its position in the device, shape and orientation), an electrostatic equation based on Maxwell–Ampere's law is solved to compute the potential $\psi_{\mathrm{RBC}}$. The cell‐free potential $\psi_{\mathrm{CF}}$ is also computed:

(7) $$\nabla \cdot \left(\sigma_{\mathrm{ext}}\nabla \psi_{\mathrm{CF}}\right)=0,$$

(8) $$\nabla \cdot \left(\sigma_{\mathrm{var}}\left(t\right)\nabla \psi_{\mathrm{RBC}}\left(t\right)\right)=0.$$

$\sigma_{\mathrm{ext}}$ is the conductivity of the suspending medium. $\sigma_{\mathrm{var}}\left(t\right)$ is the time‐dependent conductivity field, equal to $\sigma_{\mathrm{ext}}$ outside the RBC and $\sigma_{\text{in}}$ inside. Once Equations (7) and (8) are solved, the potential gradient is computed to obtain the electric field $\vec{E}$. Then, $\vec{E}$ is integrated over the orifice surface entrance S to calculate the resistance R, knowing the applied potential between the two electrodes, U:

(9) $$R=\frac{U}{\int_{S}\sigma \vec{E}\cdot d\vec{S}},\;\text{with}\;\vec{E}=\nabla \psi .$$

##### 2.1.1.3 Parametric Study

In this study, the device and its operating conditions are fixed, with unchanged geometry, flow rate, and electrode potential. A sensitivity analysis is performed to examine how RBC geometry, mechanical properties, and position/orientation affect the results. Simulations use RBC properties characteristic of healthy cells, Table 1 providing typical parameter ranges from the literature.

**TABLE 1:** Typical values of morpho‐rheological properties for healthy RBCs, found in the literature, along with possible ϕ‐orientations in the CC.

Parameter name	Parameter bounds ([min, max])	Tested values
Shear modulus $G_{s}$ (μN.m^−1^) [7, 8, 10]	[2.2, 22.5]	2.2, 12.4, 22.5
Internal visosity $\nu_{\text{in}}$ ($10^{-6}$ m^2^.s^−1^) [41, 42, 43]	[7.0, 15.0]	7.0, 11.0, 15.0
Sphericity index Q [44, 45, 46]	[0.57, 0.78]	0.57, 0.68, 0.78
Membrane viscosity $\eta_{\text{memb}}$ (Pa.m.s) [29, 31, 47]	[3.0 × 10^−8^, 1.0 × 10^−6^]	0, 5.0 × 10^−9^, 2.5 × 10^−8^
Change area modulus $E_{a}$ (N.m^−1^) [19, 25, 48]	[0.238, 0.338]	0.25
Bending modulus $E_{b}$ (J) [19, 25, 49]	[3.0 × 10^−19^, 8.0 × 10^−19^]	6.0 × 10^−19^
ϕ orientation	[0, $\frac{\pi }{2}$]	0, $\frac{\pi }{4}$, $\frac{\pi }{2}$

*Note:* The values tested in the parametric study are also specified.

In the industrial device, the micro‐orifice is cylindrical and the flow field is axisymmetric around the central axis of the orifice [19]. Without loss of generality, we consider an RBC whose center of mass lies in the ($\vec{x}$, $\vec{y}$) plane shown in Figure 1a. In this study, we will consider a unique trajectory to focus our attention on the other parameters of the RBC (orientation, shape and mechanics). The dependence of the pulse on the trajectory has been studied previously [19, 25].

**FIGURE 2:**
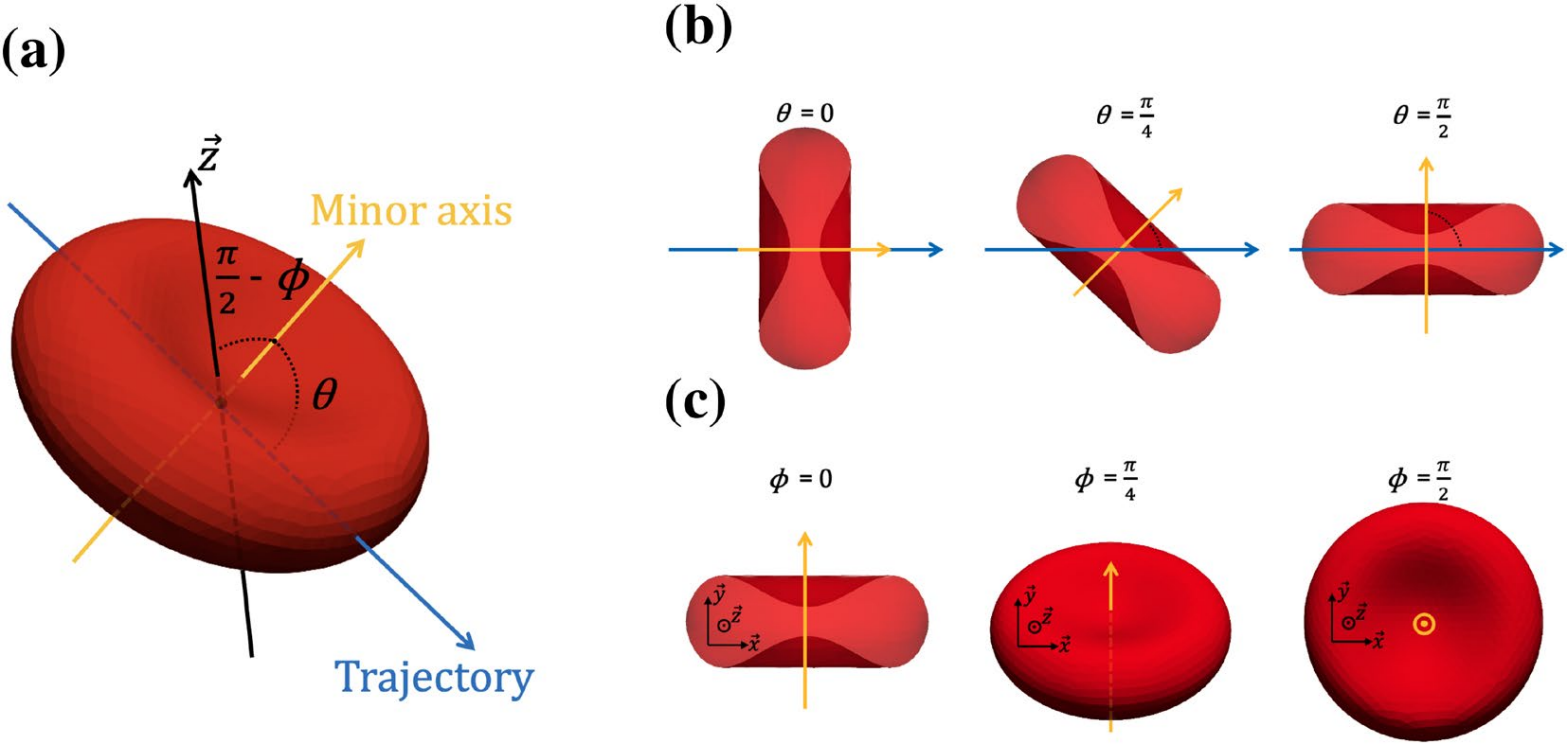
Discocyte RBCs with different orientation. The orientation of an RBC is characterized by θ and ϕ, the angle of the RBC minor axis relative to the RBC trajectory, and the angle relatively to the $\left(\vec{x},\vec{y}\right)$ plane. (a) An RBC with a sphericity index Q=0.65. The yellow arrow designates the RBC minor axis. The blue arrow corresponds to the RBC trajectory, while the black arrow designates $\vec{z}$ axis from the coordinate system presented Figure 1a. (b) RBCs with various θ orientation. (c) RBCs with various ϕ orientation.

Across the RBC tank (upstream the orifice), the RBCs are subjected to axisymmetric extensional flow along their trajectory [19, 20]. The RBCs are reoriented by the flow and their minor axis, shown in Figure 2a, tends to be orthogonal to the trajectory before entering the orifice [20, 50]. The initial angle between the RBC minor axis and its trajectory in the RBC tank, denoted by θ, is illustrated in Figure 2a,b. Due to the reorientation by the flow, it is assumed to have negligible effect on pulses and is therefore initialized to $\frac{\pi }{2}$. Except for θ orientation, the upstream flow does not impose any other constraint on RBC orientation. Therefore, the initial angle formed between the RBC minor axis and the plane $\left(\vec{x},\vec{y}\right)$, called ϕ, has been included in the factors that may impact RBCs dynamics in the orifice. Figure 2a,c illustrate RBC ϕ orientation. Note that ϕ was not mentioned in the work of Taraconat et al. [25], in which it had been fixed to 0.

In the simulations, we fixed $S_{\mathrm{RBC}}$ to a typical value of 133.4 μm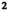. The volume of the RBC then varies linearly with Q. The mechanical moduli $E_{a}$ and $E_{b}$ have been set to 0.25 N.m^−1^ and $6.0\times 10^{-19}$ J, respectively. The spontaneous curvature of RBC membrane has been fixed to $C_{0}=0$ m^−1^ in this study. Varying this value could be particularly interesting for studying non‐discocyte RBC shapes, such as stomatocytes or echinocytes [51].

A numerical parametric study was conducted involving $G_{s}$, $\nu_{\text{in}}$, Q (and thus the RBC volume), ϕ, and $\eta_{\text{memb}}$. For each parameter, three levels were defined representing the lowest, mid‐range, and highest values reported in the literature for healthy RBCs (see Tested values in Table 1). Note however that the range of $\eta_{\text{memb}}$ from the literature is very large, spanning two orders of magnitude. We decided to explore several values and present the results for $\eta_{\text{memb}}=0$ (no membrane viscosity), $5\times 10^{-9}$ Pa.m.s, and $25\times 10^{-9}$ Pa.m.s. It is worth noting that the highest value tested is lower than those reported for normal RBCs in the literature. This will be further commented in Section 4.

#### 2.1.2 Meshing and Dynamic Mesh Adaptation

As already mentioned, the surface area of the RBC membrane is here a fixed parameter: $S_{\mathrm{RBC}}=133.4$ μm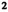. The membrane is discretized with elements of size $h_{\mathrm{RBC}}=0.3$ μm, (which corresponds to approximately 4000 faces and 2000 nodes for one RBC). In the IBM, the best accuracy is obtained when the fluid grid size is similar to that of the membrane [26]. As a consequence, the fluid grid size near the cell must be approximately 0.3 μm. Unstructured grids allow to coarsen the grid far from the cell. In previous works [19, 25], the fluid grid size was set to $h_{\mathrm{RBC}}$ over the whole RBC trajectory and this grid was static during the whole simulation.

We introduce a dynamic MA method for simulating RBC dynamics in the CC, which refines the grid locally around the RBC as it moves. The method relies on remeshing: the fluid grid is held fixed until a specified criterion is reached, at which point a new grid is generated with a prescribed mesh size across the domain. We first detail how the mesh size is defined at remeshing, and then present the criterion used to trigger the MA process.

##### 2.1.2.1 Theoretical Definition of the Mesh Size

**FIGURE 3:**
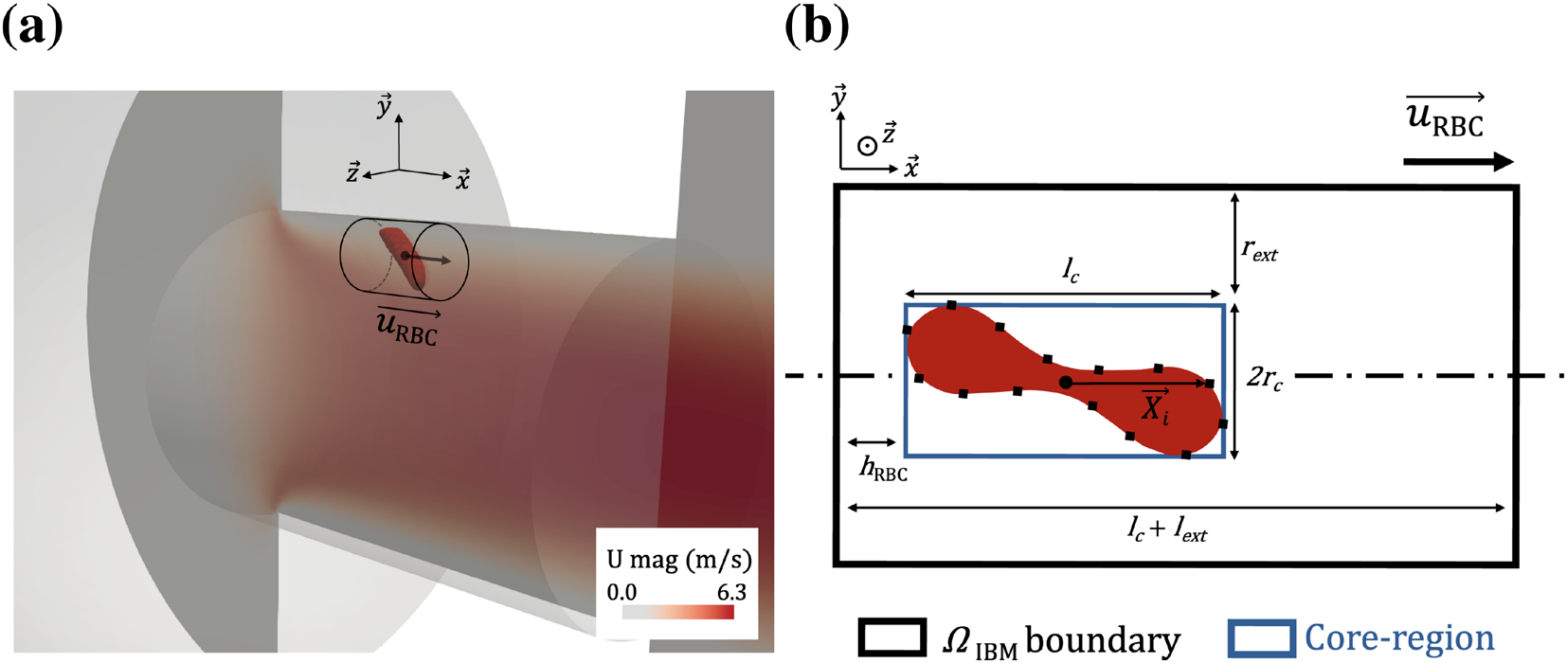
Illustration of the fine region $\Omega_{\mathrm{IBM}}$. (a) Example of $\Omega_{\mathrm{IBM}}$ region with a black cylinder for an elongated RBC in a micro‐orifice (colored with the fluid velocity field). Black arrows designate then $\vec{u_{\mathrm{RBC}}}$ direction. (b) Schematic example (in 2D) of $\Omega_{\mathrm{IBM}}$ detailing its structure. $\Omega_{\mathrm{IBM}}$ boundary is delimited with a black rectangle. The blue rectangle delimits the core‐region, it is the smaller cylinder that encloses the RBC (aligned with $\vec{u_{\mathrm{RBC}}}$). The core region is built around an RBC whose centroid is designated by a black circle and the membrane grid nodes are depicted with a black square.

The method starts from a baseline grid, obtained by mesh convergence for the cell‐free fluid flow, with grid size $h_{0}\left(\vec{x}\right)$. In this work, $h_{0}\left(\vec{x}\right)$ is larger then $h_{\mathrm{RBC}}$, the membrane mesh size and target grid size in the IBM framework. We thus define a volume $\Omega_{\mathrm{IBM}}$ around the RBC, within which the grid size $h_{\mathrm{RBC}}$ is imposed. This volume is chosen to be cylindrical. Figure 3a displays $\Omega_{\mathrm{IBM}}$ in an example, with its characteristics detailed later. Once $\Omega_{\mathrm{IBM}}$ is defined, the target grid size is expressed as a function of $h_{\mathrm{RBC}}$, $h_{0}\left(\vec{x}\right)$ and a blending function $\alpha \left(\vec{x}\right)$:

(10) $$\begin{array}{c} h\left(\vec{x}\right)=\alpha \left(\vec{x}\right)\;h_{\mathrm{RBC}}+\left(1-\alpha \left(\vec{x}\right)\right)h_{0}\left(\vec{x}\right),\\ \;\text{with}\;\left\{\begin{array}{cc} \alpha \left(\vec{x}\right)=1, & \text{if}\;\vec{x}\in \Omega_{\mathrm{IBM}},\\ \alpha \left(\vec{x}\right)\in [0,1]\;\text{such that}\;\nabla h\left(\vec{x}\right)<\nabla h_{\max}, & \text{if}\;\vec{x}\notin \Omega_{\mathrm{IBM}} \end{array}\right. \end{array}$$

Besides applying a mesh size of $h_{\mathrm{RBC}}$ within the refined region $\Omega_{\mathrm{IBM}}$ (where $\alpha \left(\vec{x}\right)=1$), $\alpha \left(\vec{x}\right)$ must be kept as close to 0 outside $\Omega_{\mathrm{IBM}}$, so that the mesh size tends toward the background mesh size $h_{0}\left(\vec{x}\right)$. However, $\alpha \left(\vec{x}\right)$ is modulated by the gradient of the mesh size $h\left(\vec{x}\right)$ to avoid abrupt variations in element size. Following Grenouilloux et al. [52], the mesh gradient $\nabla h\left(\vec{x}\right)$ must not exceed a maximum threshold $\nabla h_{\max}=0.3$. Accordingly, $\alpha \left(\vec{x}\right)$ is defined as the smallest positive value that ensures the condition $\nabla h\left(\vec{x}\right)\leq 0.3$ throughout the domain.

Naturally, $\Omega_{\mathrm{IBM}}$ must fully encompass the RBC. Moreover, to minimize the frequency of remeshing, this region is extended to include the cell's anticipated future positions. To achieve this, we assume that the RBC's future positions can be reasonably estimated using its mean velocity at the time of remeshing, $\vec{u_{\mathrm{RBC}}}$. We first define the so‐called core region as the smallest cylinder, with its axis aligned with the cell's mean velocity, that fully encloses the RBC. Its axis length and radius read:

(11) $$l_{c}=\max_{i}\;\vec{{\hat{u}}_{\mathrm{RBC}}}\cdot \vec{X_{i}}\;+\;|\;\min_{i}\;\vec{{\hat{u}}_{\mathrm{RBC}}}\cdot \vec{X_{i}}\;|$$

(12) $$r_{c}=\max_{i}\;\sqrt{{\left\|\vec{X_{i}}\right\|}^{2}-{\left(\vec{{\hat{u}}_{\mathrm{RBC}}}\cdot \vec{X_{i}}\right)}^{2}}$$

$\vec{X_{i}}$ designates the vector connecting the RBC centroid to the membrane grid node $X_{i}$, and $\vec{{\hat{u}}_{\mathrm{RBC}}}$ is a unit vector directed by $\vec{u_{\mathrm{RBC}}}$.

That cylinder is then extended in the direction of the velocity vector by a length $l_{\mathrm{ext}}$. Additionally, because the RBC is generally subjected to shear‐induced rotation, the cylinder's radius is further extended by $r_{\mathrm{ext}}$ to ensure that the cell remains within the fine region despite rotational motion. This prevents premature exit from the refined mesh zone. Figure 3b illustrates $\Omega_{\mathrm{IBM}}$ sizing details with the core‐region bounded by a blue rectangle, and the entire fine region bounded with a black rectangle. Note that $l_{\mathrm{ext}}$ and $r_{\mathrm{ext}}$ are user‐defined parameters. Their effect will be tested in Section 3.1.

##### 2.1.2.2 Practical Definition of the Mesh Size for the Use of the Remeshing Library MMG3D

In order to perform the MA, YALES2BIO is coupled with MMG3D [53, 54], a library for the adaptation and the optimization of tetrahedral meshes. MMG3D remeshes the existing grid using a metric field, which, in the case of isotropic meshing, defines the local target element size. This metric field is defined at each node of the previous fluid grid. In order to impose the grid size $h_{\mathrm{RBC}}$ in the fine region, it is prescribed that for all grid elements intersecting the cylinder describing the fine region, the vertices of the elements have a metric value of $h_{\mathrm{RBC}}$. The edge lengths of the grid are adjusted by MMG3D to match the desired size, either through edge splitting or collapsing, or by inserting nodes followed by an update of the connectivity. Further information on the MMG3D remeshing algorithm and its integration into YALES2BIO can be found in the relevant literature [52, 53, 54, 55].

##### 2.1.2.3 Criterion for Remeshing

The criterion that governs when the remeshing process is triggered remains to be specified. At each iteration, the positions of all membrane mesh vertices are compared with the boundaries of the fine region defined during the previous remeshing. If any membrane node lies within a distance less than $h_{\mathrm{RBC}}$ from the boundary of this region, a new fine region is then defined around the RBC and remeshing is triggered. This ensures that the RBC remains entirely within a portion of the grid that satisfies the resolution required by the IBM. The new fine region necessarily overlaps with the previous one. In this overlapping region, the existing mesh already satisfies the prescribed remeshing metric. The remeshing algorithm is instructed to preserve the previous grid in this area, so that no interpolation is required in the critical region surrounding the RBC.

##### 2.1.2.4 Remeshing Algorithm

The remeshing algorithm can be summarized as follows. It starts from a grid with an associated fine region $\Omega_{\mathrm{IBM}}$ and a given RBC position.

1. At each iteration, the FSI system is solved, and the RBC position is updated
2. A remeshing trigger test is performed to determine whether the RBC is close enough to the boundaries of the fine region $\Omega_{\mathrm{IBM}}$. If not, the next iteration proceeds. Otherwise, remeshing is initiated.
3. A new fine region is defined by first calculating the center of mass and the mean velocity of the RBC. The core region of length $l_{c}$ and radius $r_{c}$ is constructed around the RBC. This core is then extended based on user‐defined parameters $l_{\mathrm{ext}}$ and $r_{\mathrm{ext}}$ to form the new fine region.
4. The remeshing metric is assigned to all vertices of the existing grid, based on whether their associated elements lie within the new fine region. For all grid nodes located in the overlapping area between the previous and the new fine region, the mesh is frozen to avoid modification.
5. The metric field over the entire fluid grid is iteratively modified so that $\nabla h\left(\vec{x}\right)<\nabla h_{\max}$.
6. The MMG3D library is called to perform remeshing, and the solution from the old grid is interpolated onto the new one. The next iteration proceeds.

In Section 3.1, results obtained with and without MA are compared, and the computational cost savings are evaluated as a function of the remeshing algorithm's free parameters, $l_{\mathrm{ext}}$ and $r_{\mathrm{ext}}$.

#### 2.1.3 Numerical Pipeline: From the Input Parameters to the Resistive Pulse

##### 2.1.3.1 Simulations Without RBCs

From the industrial geometry shown in Figure 1a, the fluid domain is defined. It includes the RBC tank, the micro‐orifice and the exit tube. A constant flow rate of $7.74\times 10^{-9}$ m
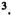
s^−1^ is imposed at the inlet to replicate the pressure differential applied in the ABX Micros 60. The electrolyte classically used in the ABX Micros 60 is mainly composed of water. Therefore, we set $\nu_{\mathrm{ext}}=1.0\times 10^{-6}$ m
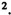
s^−1^ and ρ=1000 kg.m^−3^. In addition, the electric potential without cell is computed to assess the resistance of the system in the absence of RBC, $R_{\mathrm{CF}}$. The conductivity of the electrolyte is set to $\sigma_{\mathrm{ext}}=2.27$ S.m^−1^. A potential of U=13.9 V is applied between the electrodes, as in the ABX Micros 60. Other walls are assumed to be insulating.

To compute the carrying flow in the cell‐free configuration, the grid is set with a mesh size of 1.6 μm in the micro‐orifice region, while the mesh size gradually increased with a growth rate of 1.3 in the rest of the configuration, so that mesh size in the coarser region of the domain reach 500 μm. The total number of elements is approximately 3.0$\times 10^{7}$.

##### 2.1.3.2 Deformation of the RBC in the Tank Before Reaching the Orifice (FSI, Phase I)

The output of interest of the CC simulations is the pulse associated with the passage of an RBC in the micro‐orifice, which depends on the RBC deformation in the orifice itself. However, in the industrial CCs, RBCs can be deformed by the flow upstream of the orifice. Neglecting this deformation leads to erroneous predictions in the orifice [19]. As a full‐scale simulation of the RBC dynamics within the entire device would be computationally expensive and impractical, the RBC deformation by the flow before the orifice is predicted in a first FSI simulation, referred to as phase I. This approach has been validated by Taraconat et al. [19].

**FIGURE 4:**
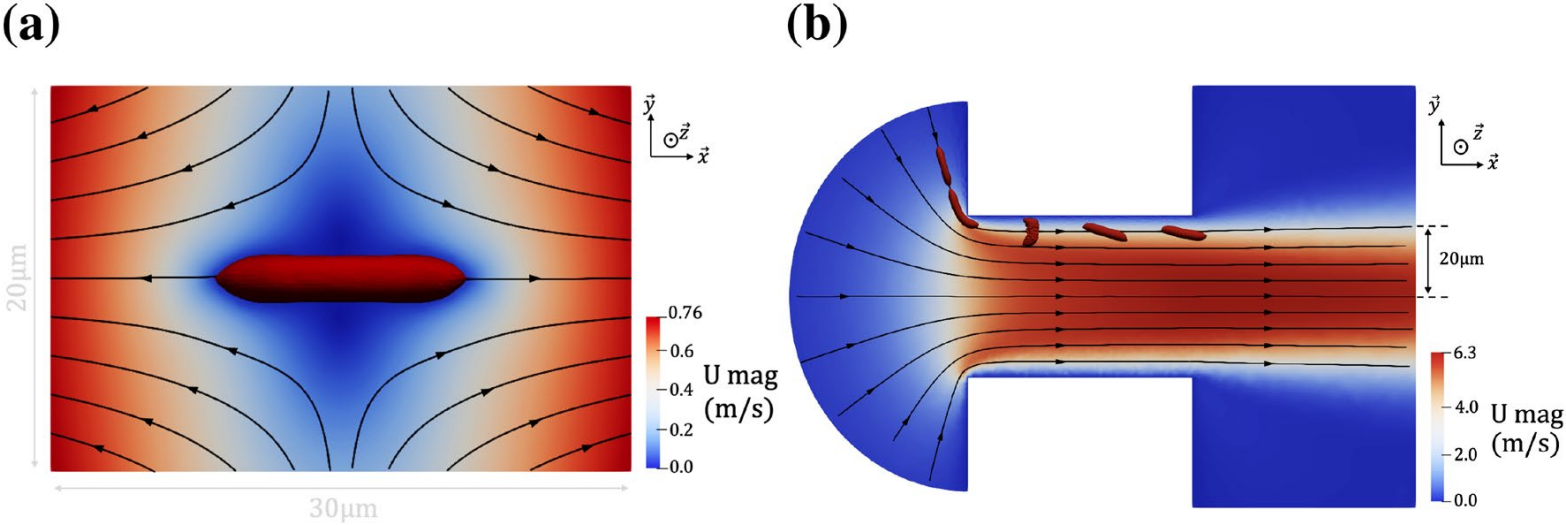
The two phases FSI calculation. (a) Phase I: Slice of the domain in which the deformation of the RBC upstream of the micro‐orifice is mimicked by applying the correct strain‐rate tensor along time. The colormap shows the fluid velocity magnitude. The flow is extensional and is outlined with black streamlines. The boundary conditions are deduced from the strain‐rate tensor experienced by an RBC from the bulk of the tank until ≈10 μs before it would enter the orifice. (b) Phase II: Slice of a small portion of the domain around the orifice. The colormap represents the fluid velocity magnitude in the absence of RBC. Streamlines are outlined in black. An example of RBC dynamics near the orifice wall is superimposed by displaying the shape of the membrane at five instants. The initial RBC state for that calculation (phase II) is the last RBC state of the calculation presented Figure 4a (phase I).

Instead of predicting the RBC deformation in the full system, it is calculated in a small domain, by applying the same strain‐rate tensor history as in the real system. In this phase I calculation, the RBC is fixed, aligned with the strain direction, and subjected to a pure elongational flow representative of the conditions experienced upstream of the aperture. Velocity gradients are extracted along a streamline—taken as a good approximation of the RBC trajectory—from the cell‐free simulation, and imposed as time‐dependent Dirichlet boundary conditions on a cylindrical domain. This is illustrated in Figure 4a, in which the streamlines are characteristic of a purely linear extensional flow, in the frame of the RBC. For the FSI calculations, the IBM requires that the fluid grid resolution be comparable to the solid grid size $h_{\mathrm{RBC}}$ [26]. In Phase I simulation, the RBC's center of mass remains approximately centered within the domain. Accordingly, a mesh size of $h_{\mathrm{RBC}}=0.3$ μm was applied within a spherical region of radius $=\sqrt{\frac{S_{\mathrm{RBC}}}{\pi }}$ (twice the length of the longest axis of the RBC at rest) centered at the domain centroid. Outside this region, the mesh size was set to 1.0 μm, resulting in a total of approximately $6\times 10^{5}$ elements.

While the time‐history of the velocity gradient only depends on the trajectory, which is fixed in this study, this calculation has to be performed for each set of RBC parameters. The resulting RBC state serves as the starting configuration for the RBC in phase II.

##### 2.1.3.3 Deformation of the RBC in the Orifice (FSI, Phase II)

Phase II focuses on the RBC dynamics within the orifice, with the computation restricted to a portion of the industrial geometry surrounding the orifice (see Figure 4b). The constant flow rate in the entire device also passes through the orifice. It is imposed at the inlet of the domain (left of Figure 4b). The RBC is deposited before the orifice in its deformed state after Phase I calculation. In the orifice, the trajectory is such that the RBC flows at 5 μm from the wall and 20 μm from the orifice axis.

In the FSI Phase II domain, elements of size $h_{\mathrm{RBC}}$ are restricted to a limited zone around the cell, using dynamics mesh adaptation as described in Section 2.1.2. Outside this refined region, the mesh size was set to 1.0 μm in the orifice area, while the mesh size gradually increased with a growth rate of 1.3 in the rest of the configuration, so that mesh in the coarsest region of the domain reaches 5 μm. The total number of elements for this configuration is approximately 1.3$\times 10^{7}$.

##### 2.1.3.4 Impedance Pulse

The impedance pulse is obtained by computing a series of electrostatic problems to determine the additional resistance of the system due to the RBC. An accurate description of a pulse is achieved by computing this resistance every microsecond. Results from phase II FSI simulations are thus stored every microsecond and used as an input of the electrostatic calculations. The same boundary conditions as in the electrostatic cell‐free simulation are applied. However, the mesh used here differs due to the presence of an RBC, which requires finer elements of size $h_{\mathrm{RBC}}$ in the region occupied by the RBCs.

##### 2.1.3.5 Computing Resources, and Calculation Cost

Each simulation was conducted on one node with 128 cores. Each compute node is equipped with dual AMD EPYC 7xx2 series processors, running at 2.6 GHz and 256 GB of RAM. Using the dynamics remeshing for phase II, calculations in phase I, phase II, and the series of electrostatic calculations take approximately the same CPU time. The total computational cost was approximately 600 CPU hours for one pulse, corresponding to slightly less than 5 h of wall‐clock time.

### 2.2 Experimental Set‐Up

#### 2.2.1 Sample and Instrument

This study uses nine human healthy blood samples collected during routine medical care and provided anonymously to HORIBA in compliance with ethical and regulatory standards. The blood samples were analyzed less than 6 h after collection using the ABX Micros 60 from HORIBA. It features an enhanced bandwidth of 150 kHz to minimize signal distortion caused by the electronics in the standard commercial model [19, 25]. Upon insertion of the sample tube, a needle withdraws an aliquot and transfers it to the RBC tank. The sample is then diluted 1/15000 in a phosphate buffered saline (PBS) electrolytic reagent, formulated with an osmolarity of 297 mOsm. A vacuum pump then aspirates the suspension through a micro‐orifice with a 200 mbar pressure drop across the aperture. The geometry of the RBC tank and of the micro‐orifice are sketched in Figure 1a,b.

During vacuuming, a constant electric current is applied using the two electrodes. Note that the electric conditions applied differ from the standard experimental setup of the ABX Micros 60 (described in Taraconat et al. [25]). Indeed, a PBS reagent has been used as it is presumed not to alter RBCs properties, but its conductivity (approximately 2.7 S.m^−1^ at 35°) is about 1.2 times greater than the standard reagent. Moreover, there were concerns about electroporation effects at the typical electric current level [56]. As a result, the electric current was reduced by a factor of 1.3, resulting in a steady current of 0.41 mA, which was chosen to maintain an optimal signal‐to‐noise ratio.

Pulses are recorded thanks to an in‐house Lab‐VIEW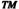 (National Instruments) code [19]. During the recording, the raw experimental signatures undergo modifications by the ABX Micros 60 hardware: amplified for ensuring their usability in the rest of the system and then edited with a bandpass filter to minimize the noise.

#### 2.2.2 Pulse Selection for Comparison

**FIGURE 5:**
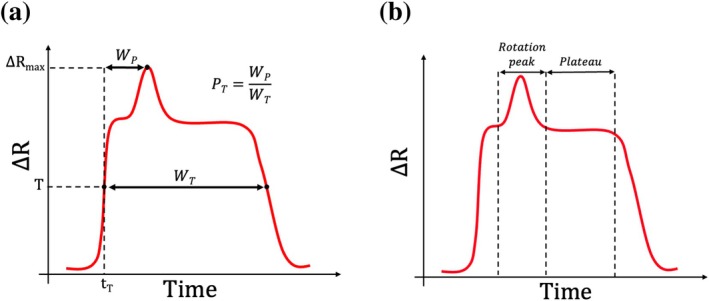
Characterization of impedance pulses. The red curves represent schematic impedance signatures. (a) Illustration of signatures characteristics $W_{T}$ and $P_{T}$ introduced for filtering experimental signatures by trajectory. (b) Delimitation of signature zones of interest for inspecting the effect of RBC deformability.

As stated in Section 2.1.1, the study focuses on a single trajectory. Ideally, the numerical results should be compared to experimental pulses for the same trajectory. However, it is impossible to directly determine the trajectory associated to a pulse in the experiments. However, we can select experimental pulses that share some specific features with the simulations, which are strongly correlated with the trajectory. As demonstrated in the work of Taraconat et al. [19], the RBC trajectory influences the pulse width and the time at which the rotation‐peak is generated: when the RBC flows closer to the orifice wall, its velocity decreases, resulting in a longer pulse duration; in addition, the shear rate (here the gradient of axial velocity with respect to the direction normal to the wall) is higher, causing the RBC to rotate earlier. Two pulse characteristics, measuring the pulse duration and the rotation‐peak period, are therefore used for pulse selection, and defined in Figure 5a.

$W_{T}$ is the width of the pulse above a threshold T: it is the time during which ΔR≥T. $W_{T}$ informs about the time taken by an RBC to flow through the micro‐orifice. $P_{T}$ is the rotation‐peak relative position, calculated as: $P_{T}=\frac{W_{P}}{W_{T}}$, with $W_{P}$ the time delay between the moment when the signal exceeds the threshold T (t = $t_{T}$) and the moment when the signal reaches its maximum $\Delta R_{\max}$, which generally corresponds to the rotation peak. Note that T is set to 50% of $\Delta R_{\max}$ [25]. $P_{T}$ characterizes the moment when the cell rotates in the micro‐orifice. The smaller $P_{T}$, the earlier the rotation. Since they are partly controlled by the RBC trajectory, these two metrics are used in Section 3.3 for filtering experimental signature by trajectory. To filter the experimental pulses, the minimum and maximum values of $W_{T}$ and $P_{T}$ were assessed across all simulated signatures, and any pulses outside these established limits were discarded. Obviously, this technique is an approximation of a trajectory selection. From previous results, we estimate that the uncertainty in the radial position of RBC passage is below 5% of the aperture radius.

Hardware modifications applied to experimental pulses (c.f. Section 2.2.1) are not modeled in the simulations. Indeed, the gain applied to experimental pulses adjusts to compensate for the effects induced by temperature variation and has not been measured during data acquisition. Direct comparison between simulated and experimental results is therefore impossible. To overcome this issue, both numerical and experimental pulses are expressed in terms of resistive perturbation, then normalized with a pulse‐dependent scaling factor. This factor is equal to the “plateau” amplitude illustrated in Figure 5b. As previously stated, the main effects of deformability on pulses are expected in the rotation peak region. Therefore, scaling pulses by the plateau amplitude emphasizes the effects of RBC deformability on pulses. The plateau amplitude is computed at time $t=t_{T}+0.70W_{T}$, as this is a moment when the pulse's slope from simulated data (presented in Sections 3.2, 3.3) is the closest to zero on average. The notation ΔR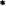 denotes the scaled amplitude of the numerical pulses. This normalization strategy also mitigates the influence of RBC volume on the pulse signals, thereby emphasizing the other RBC properties [25].

## 3 Results

### 3.1 Numerical Approach Validation and Optimization

In this section, the reliability of the numerical approach and the efficiency of the mesh adaptation strategy are assessed. First, a simulation was performed using a static fluid grid, which serves as a reference: instead of using dynamic remeshing, the fluid grid is refined all along the trajectory to ensure good accuracy of the IBM without needing to remesh. This correspond to the previous state of the art in CC simulations [19, 25]. Subsequently, multiple simulations were conducted to assess the impact of the user‐defined parameters ($l_{\mathrm{ext}}$ and $r_{\mathrm{ext}}$) that control the size of the refinement region in the dynamic remeshing. Nine simulations with MA were performed, combining three values for both $r_{\mathrm{ext}}$ and $l_{\mathrm{ext}}$: 1 μm, 5 μm, and 10 μm.

**FIGURE 6:**
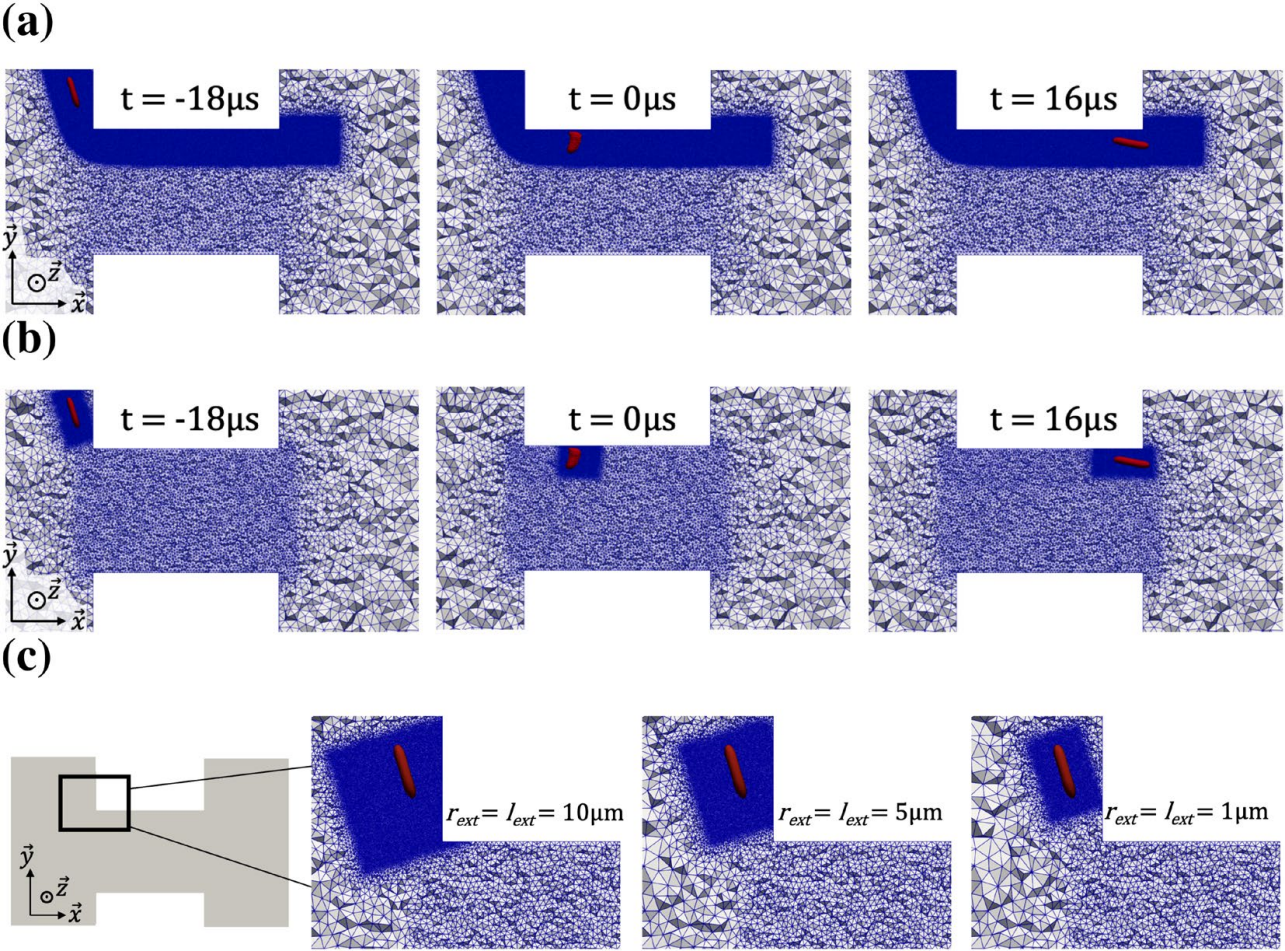
Simulations of the FSI calculation phase II, with and without MA. (a) RBC states and fluid grids at different instants in a simulation performed with a static fluid grid. The times reported are shifted so that t = 0 when the RBC is rotating and the associated pulses reaches its maximum amplitude. At *t* = −18 μs, the RBC is too far from the aperture entrance and the electric perturbation of the RBC is negligible. At *t* = 16 μs, the RBC is exiting the aperture and the resisitive perturbation decreases (descending slope after plateau region). (b) RBC states and fluid grid states at different instants in a simulation with MA ($l_{\mathrm{ext}}=5$μm, $r_{\mathrm{ext}}=1$ μm). (c) Examples of MA cases performed with different $\Omega_{\mathrm{IBM}}$ size, focusing on the upper area at the orifice entrance. The grids are obtained at initialization (*t* = −18 μs) of FSI phase II calculation.

The RBC parameters used in this study are: $G_{s}$ = 2.5 μN.m^−1^, $E_{a}$ = 2.5 × 10^−1^ N.m^−1^, $E_{b}$ = 6.0 × 10^−19^ J, Q = 0.65, $\nu_{\text{in}}$ = 18.0 × 10^−6^ m^2^.s^−1^, $\eta_{\text{memb}}$ = 0 and ϕ = 0. Figure 6a and Figure 6b illustrate simulation results obtained at different time instants using a static fluid grid and a dynamically adapted grid with $l_{\mathrm{ext}}=5$ μm and $r_{\mathrm{ext}}=1$ μm, respectively. Figure 6c presents examples of grids generated during the initialization for various sets of $l_{\mathrm{ext}}$ and $r_{\mathrm{ext}}$.

The resulting RBC dynamics (not shown here) are very similar across all cases. Before entering the orifice, the RBC undergoes significant elongation and maintains this elongated shape throughout its passage. It rotates during the first third of the orifice, then gradually realigns with the aperture axis before exiting.

**FIGURE 7:**
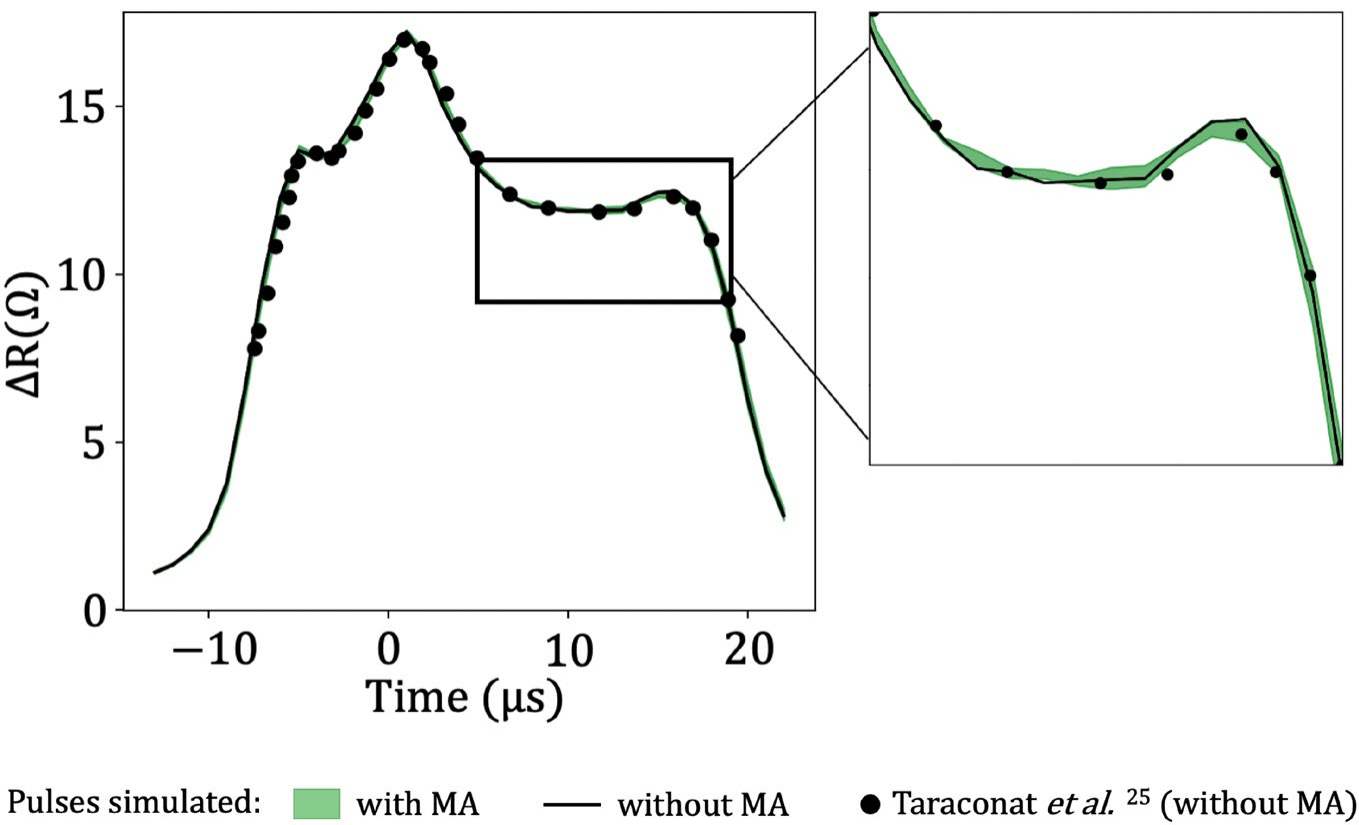
Impedance signatures resulting from simulations with and without mesh adaptation. The pulses obtained with MA are enclosed in a green envelope indicating the minimum and maximum values of ΔR at each time instant, over all cases. The pulse obtained using a static fluid grid is shown as a solid black line. Black dots represent a reference pulse previously obtained under the same conditions with a static grid, as reported in Taraconat et al. [25].

Figure 7 shows the corresponding resistance pulses. All cases performed with MA are represented by a green envelope indicating the minimum and maximum ΔR values at each time instant. The result for the case without MA is represented by a solid black line. Additionally, results obtained by Taraconat et al. [25] under identical conditions (same RBC, trajectory, flow parameters, and geometry), but using a different static grid, are also plotted for comparison. The green envelope closely follows the black solid line, indicating that the MA strategy yields results nearly identical to those obtained with a static grid. Furthermore, the strong agreement with numerical results from Taraconat et al. [25] (previously validated against experimental data) supports the reliability of our numerical approach. The deviation in ΔR is always less than 4%, confirming the accuracy of the simulations. MA was tested on other trajectories, with similar accuracy.

**FIGURE 8:**
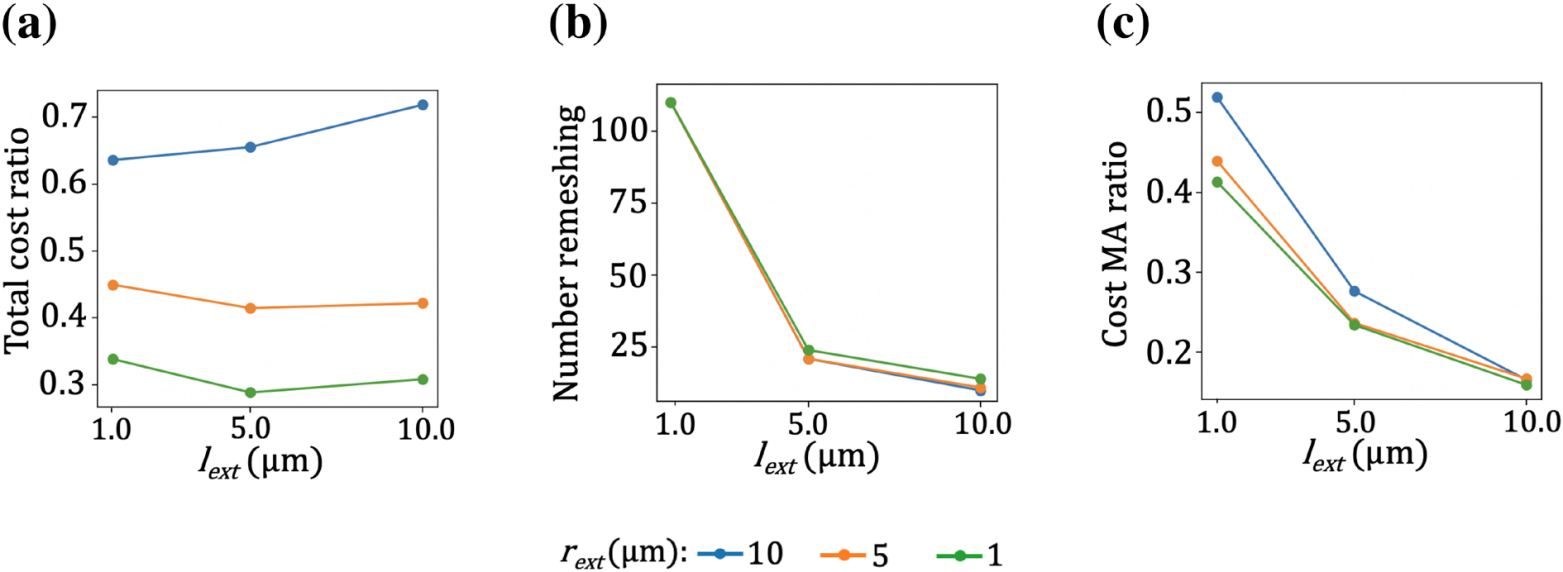
Performance metrics of the simulations using mesh adaptation during FSI calculation phase II. The results are plotted as a function of $l_{\mathrm{ext}}$ and correspond to different configurations of the fine region around the RBC. Curve colors correspond to different values of $r_{\mathrm{ext}}$. (a) Cost of each MA case, normalized by the cost of a simulation without MA. (b) Number of remeshing events triggered during the simulation. (c) Proportion of total computational cost attributed to the mesh adaptation process.

The performances are now assessed. Figure 8a shows the computational cost of each MA case, normalized by the cost of the simulation performed with the static fluid grid. For all tested combinations of $l_{\mathrm{ext}}$ and $r_{\mathrm{ext}}$, the cost ratio is less than 1, indicating that the reduction in the number of elements achieved thanks to MA effectively lowers the computational cost during the FSI phase II calculation despite the additional cost of remeshing. For reference, the grid shown in Figure 6a (without MA) contains approximately $13\times 10^{6}$ elements, whereas the dynamically adapted grid in Figure 6b averages around $2\times 10^{6}$ elements.

As shown in Figure 8a, the observed cost reductions are primarily driven by variations in $r_{\mathrm{ext}}$, as expected. The volume of the refined region is indeed proportional to ${\left(r_{c}+r_{\mathrm{ext}}\right)}^{2}\left(l_{c}+l_{\mathrm{ext}}\right)$, so changes in the radial dimension naturally have a larger impact. Furthermore, the RBC geometry yields a core region around the cell such that $r_{c}<l_{c}$, so variations in the radial extent relatively influence the refined volume more significantly than changes in length. Interestingly, the number of remeshing operations per simulation, shown in Figure 8b, is largely governed by $l_{\mathrm{ext}}$: the greater the value of $l_{\mathrm{ext}}$, the fewer remeshing operations are required. This behavior is relatively insensitive to $r_{\mathrm{ext}}$, which suggests that the optimal global performance is achieved for small values of $r_{\mathrm{ext}}$. Figure 8c further indicates that, in addition to increasing the volume of the fine region, large values of $r_{\mathrm{ext}}$ also raise the computational cost associated with remeshing. Finally, according to Figure 8a, an optimal value of $l_{\mathrm{ext}}$ appears to be around 5 μm when $r_{\mathrm{ext}}<10$ μm. This value provides a good compromise between remeshing frequency and overall simulation cost.

Among all configurations tested, the fastest simulation corresponds to $r_{\mathrm{ext}}=1$ μm and $l_{\mathrm{ext}}=5$ μm. While the original approach using a static fluid grid requires 732 h (CPUh) for this phase II calculation, the most efficient MA configuration completes the simulation in 211 CPUh. Consequently, mesh adaptation with $r_{\mathrm{ext}}=1$ μm and $l_{\mathrm{ext}}=5$ μm was used for the studies presented in Sections 3.2 and 3.3. Accounting for the whole series of calculations, mesh adaptation for phase II reduces the total cost of simulating a pulse by approximately a factor of two.

### 3.2 Influence of RBCs Properties on Pulses

In this section, the effect of RBC orientation, shape, and mechanical properties on resistance pulses is assessed using a one‐at‐a‐time (OAT) approach. From several reference parameter sets, individual parameters ($G_{s}$, $\nu_{\text{in}}$, Q, ϕ and $\eta_{\text{memb}}$) are increased, allowing their influence on the pulses to be isolated.

**FIGURE 9:**
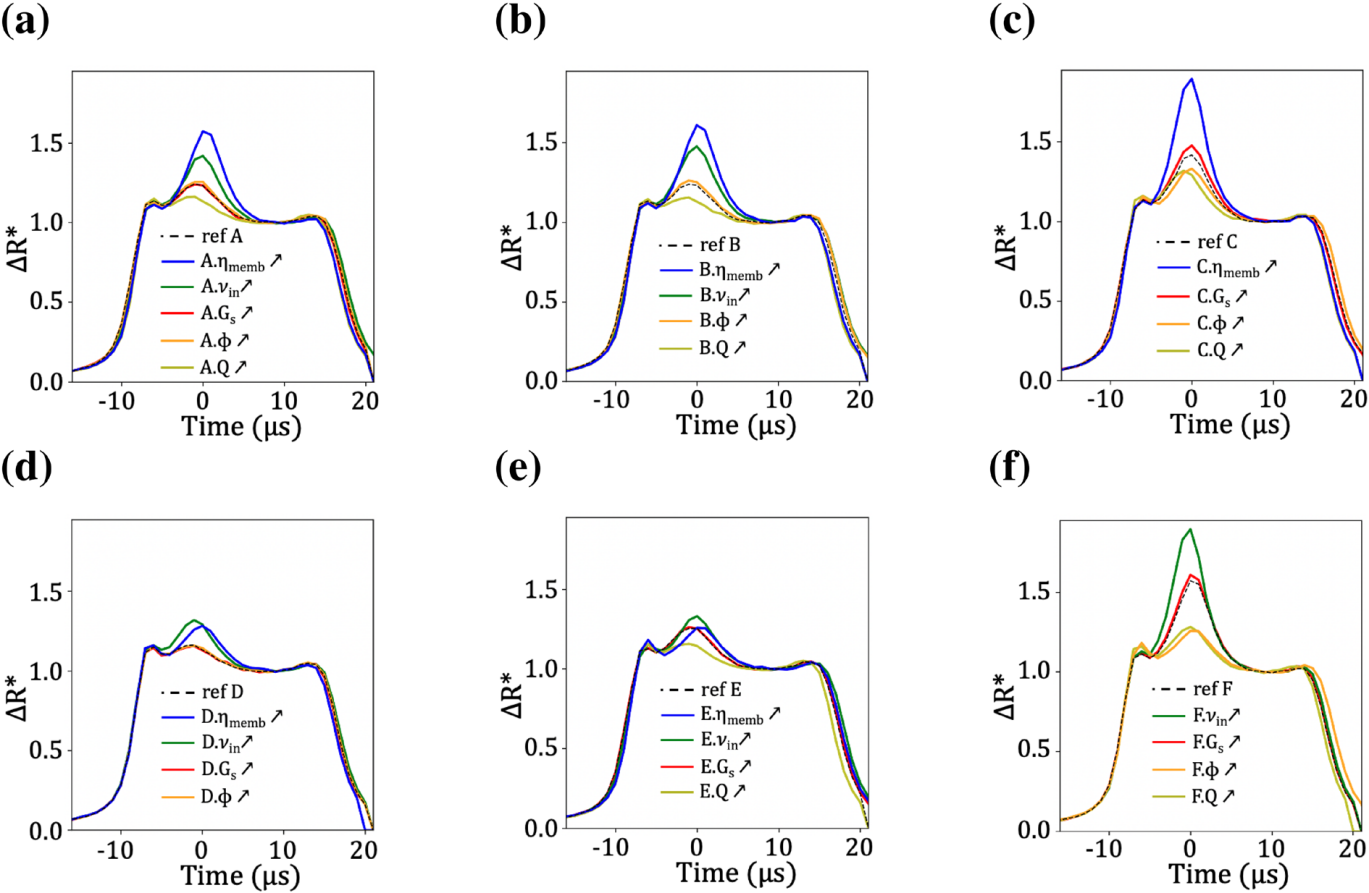
One‐at‐a‐time sensitivity analysis of the numerical pulses when varying RBC properties ($\eta_{\text{memb}}$, $\nu_{\text{in}}$, $G_{s}$, ϕ and Q). Six different operating cases were used (see the different scenarios in Table 2).

The OAT analysis was first conducted using a reference case in which all parameters were set to their lower bounds, except for Q and $\eta_{\text{memb}}$, which were fixed at intermediate values due to numerical instabilities at lower levels (see Section 3.3). From this reference case, the analysis was repeated with five alternative reference cases to test the robustness of the conclusions: in each, all parameters remained at their baseline values except one, which was increased to its maximum physiological value. The exact parameter values for all the reference cases and the sensivity analysis runs are listed in Table 2. Figure 9a–f present the resistance pulses from the sensitivity analysis. The time axis is shifted such that t=0 corresponds to the pulse's rotation peak, and the pulses are normalized (see Section 2.2.2) to eliminate the influence of RBC volume, which depends on Q.

**TABLE 2:** Overview of the cases used to study the sensitivity of pulses to different RBC properties.

Case	$G_{s}$ (μN.m^−1^)	$\nu_{\text{in}}$ ($10^{-6}$m 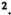 s^−1^)	Q	ϕ	$\eta_{\text{memb}}$ (10^−9^ Pa.m.s)
ref A	2.2	7.0	0.68	0	5.0
A.$G_{s}↗$	**22.5**	7.0	0.68	0	5.0
A.$\nu_{\text{in}}↗$	2.2	**15.0**	0.68	0	5.0
A.Q↗	2.2	7.0	**0.78**	0	5.0
A.ϕ↗	2.2	7.0	0.68	$\frac{\pi }{2}$	5.0
A.$\eta_{\text{memb}}↗$	2.2	7.0	0.68	0	**25.0**
ref B	22.5	7.0	0.68	0	5.0
B.$\nu_{\text{in}}↗$	22.5	**15.0**	0.68	0	5.0
B.Q↗	22.5	7.0	**0.78**	0	5.0
B.ϕ↗	22.5	7.0	0.68	$\frac{\pi }{2}$	5.0
B.$\eta_{\text{memb}}↗$	22.5	7.0	0.68	0	**25.0**
ref C	2.2	15.0	0.68	0	5.0
C.$G_{s}↗$	**22.5**	15.0	0.68	0	5.0
C.Q↗	2.2	15.0	**0.78**	0	5.0
C.ϕ↗	2.2	15.0	0.68	$\frac{\pi }{2}$	5.0
C.$\eta_{\text{memb}}↗$	2.2	15.0	0.68	0	**25.0**
ref D	2.2	7.0	0.78	0	5.0
D.$G_{s}↗$	**22.5**	7.0	0.78	0	5.0
D.$\nu_{\text{in}}↗$	2.2	**15.0**	0.78	0	5.0
D.ϕ↗	2.2	7.0	0.78	$\frac{\pi }{2}$	5.0
D.$\eta_{\text{memb}}↗$	2.2	7.0	0.78	0	**25.0**
ref E	2.2	7.0	0.68	$\frac{\pi }{2}$	5.0
E.$G_{s}↗$	**22.5**	7.0	0.68	$\frac{\pi }{2}$	5.0
E.$\nu_{\text{in}}↗$	2.2	**15.0**	0.68	$\frac{\pi }{2}$	5.0
E.Q↗	2.2	7.0	**0.78**	$\frac{\pi }{2}$	5.0
E.$\eta_{\text{memb}}↗$	2.2	7.0	0.68	$\frac{\pi }{2}$	**25.0**
ref F	2.2	7.0	0.68	0	25.0
F.$G_{s}↗$	**22.5**	7.0	0.68	0	25.0
F.$\nu_{\text{in}}↗$	2.2	**15.0**	0.68	0	25.0
F.Q↗	2.2	7.0	**0.78**	0	25.0
F.ϕ↗	2.2	7.0	0.68	$\frac{\pi }{2}$	25.0

*Note:* Each block between two horizontal lines represents a one‐at‐a‐time study conducted at a specific operating point, labeled as “ref” followed by a letter (A, B, C, D, E, or F). Relative to the operating point, individual parameters ($G_{s}$, $\nu_{\text{in}}$, Q, ϕ, and $\eta_{\text{memb}}$) are increased, and each resulting case is labeled with the same letter as the associated reference point, the modified parameter, and the symbol “↗”. For each case, the value of each parameter is provided, with the increased parameter highlighted in bold. The values $E_{a}=0.25$ N⋅m^−1^ and $E_{b}=6.0\times 10^{-19}$ J are used in all calculations.

In general, the influence of the parameters is essentially the same regardless of the chosen reference scenario. Specifically, increasing Q (yellow lines) leads to flatter pulses, whereas increasing $\nu_{\text{in}}$ (green lines) or $\eta_{\text{memb}}$ (blue lines) produces pulses with sharper, more pronounced rotation peaks relative to the reference cases (black dash lines). In the scenario where all simulations were conducted with increased sphericity (Figure 9d), these trends remain valid; however, the effect of RBC rigidity is less pronounced compared to the low‐sphericity cases. In constrast, increasing $G_{s}$ results in no significant changes compared to the reference responses, indicating that variations in shear modulus within physiological limits have a negligible effect on the pulses. This is further supported by the similarity between Figure 9a,b, where the pulses remain essentially unchanged despite a tenfold increase in $G_{s}$.

Finally, Figure 9c,f show that the influence of the initial orientation ϕ (orange lines) depends on RBC rigidity, whether controlled by internal viscosity $\nu_{\text{in}}$ or membrane viscosity $\eta_{\text{memb}}$. In the scenarios where the reference case has $\nu_{\text{in}}=7\times 10^{-6}$ m^2^.s^−1^ and $\eta_{\text{memb}}=5\times 10^{-9}$ Pa.m.s, varying ϕ has little to no effect. In contrast, when $\nu_{\text{in}}=15\times 10^{-6}$ m^2^.s^−1^ or $\eta_{\text{memb}}=2.5\times 10^{-8}$ Pa.m.s, ϕ significantly alters the pulse shape. In particular, changing ϕ from 0 to $\frac{\pi }{2}$ produces a pulse with a more subdued rotation peak. This result is illustrated in Figure 9e, where pulses simulated with $\frac{\pi }{2}$, exhibit reduced variability compared to the corresponding ϕ=0 cases shown in Figure 9a.

Overall, increasing RBC rigidity extends the range of possible rotation peak amplitudes, either preserving or enhancing the peak height depending on the parameter combination. Conversely, increasing RBC sphericity tends to reduce pulse variability in the rotation‐peak region.

### 3.3 Comparison With Experimental Data

In this section, the model's ability to reproduce the range of signatures observed experimentally is assessed (particularly in the rotation peak region). To this end, a systematic numerical parametric study was conducted involving five parameters: $G_{s}$, $\nu_{\text{in}}$, Q, ϕ, and $\eta_{\text{memb}}$. For each parameter, three levels were defined to explore the ranges of values reported for healthy RBCs (see Table 1). All possible combinations of these parameter levels were simulated, resulting in $3^{5}=243$ simulations. Although the earlier one‐at‐a‐time analysis showed that $G_{s}$ had a limited effect on the pulse shape, it was retained in this study to ensure comprehensive coverage of physiologically relevant conditions. Numerical instabilities occurred in 8 of the 243 cases (approximately 3%). These unstable cases were consistently associated with Q=0.57 and $\eta_{\text{memb}}=0$, further supporting the importance of incorporating membrane viscosity into the modeling framework.

**FIGURE 10:**
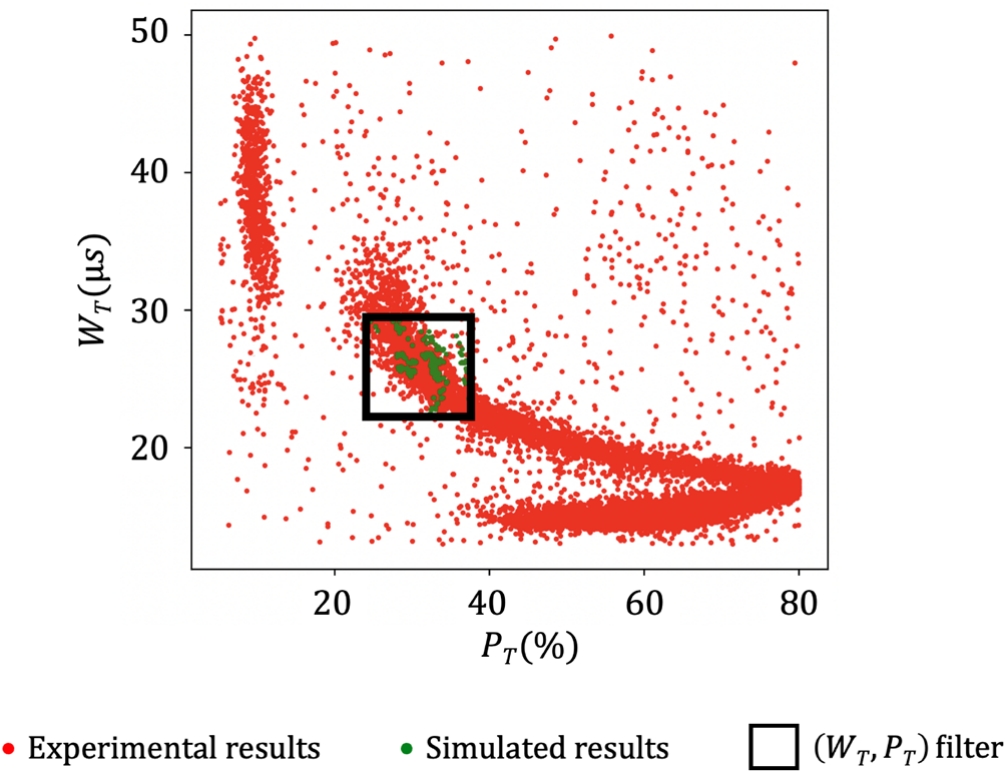
Scatter plot of experimental (red dots) and numerical (green dots) results in the ($W_{T}$, $P_{T}$) plane. The black rectangle delineates the filtering criterion used to extract experimental pulses corresponding to RBCs that followed trajectories similar to the one imposed in the simulations.

Next, data acquisition was performed on nine healthy subjects using the ABX Micros 60 analyzer, as described in Section 2.2. Approximately 15,000 pulses were recorded per acquisition. For the sake of clarity, results are shown for a single representative sample; similar results were obtained for other samples. For each of the experimental (red dots) and simulated (green dots) pulses, the metrics $W_{T}$ and $P_{T}$ are computed (Figure 5a), and the values are displayed in Figure 10. The simulated results are clustered within a confined region of the ($W_{T}$, $P_{T}$) space, where the experimental data are densely concentrated. A black rectangle indicates the filtering window applied to retain experimental pulses likely associated with RBCs following a trajectory similar to the one fixed in the simulations (see Section 2.2). Note that $W_{T}$ and $P_{T}$ results are modulated by the RBC properties and initial orientation ϕ, which explains the variability observed in Figure 10, even when simulations are conducted along a single trajectory.

**FIGURE 11:**
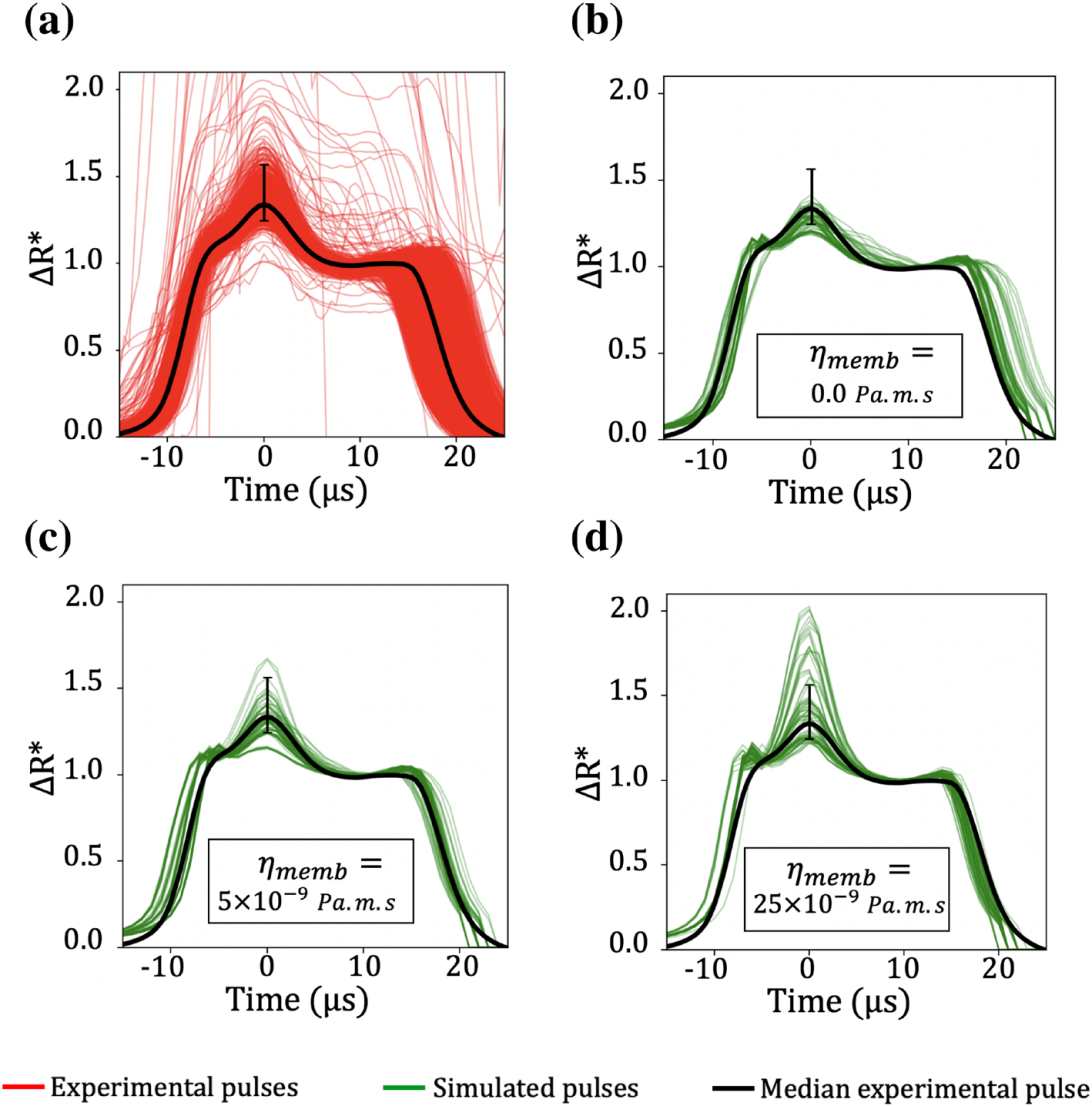
Comparison between experimental and simulated normalized pulses. All pulses are shifted in time so that their peak amplitude occurs at t=0. Panel (a) displays experimental pulses (red lines) obtained using the ABX Micros 60 on a healthy subject. The filtered experimental pulses correspond to RBCs following trajectories similar to those used in the simulations. Simulated signatures (green lines) result from a systematic parametric study. Panels (b), (c), and (d) show simulated pulses generated with $\eta_{\text{memb}}$ values of 0, $5\times 10^{-9}$ Pa.m.s, and $2.5\times 10^{-8}$ Pa.m.s, respectively. The solid black curve represents the median experimental pulse. The vertical black segment marks the rotation peak amplitude range of the experimental signatures, defined by the 5th to 95th percentiles.

The filtered experimental signatures and the simulated signatures are presented in Figure 11a–d, with green and red solid lines, respectively. Simulation results are presented by grouping the results by the value of $\eta_{\text{memb}}$ imposed: Figure 11b–d show simulated pulses generated with $\eta_{\text{memb}}$ values of 0, $5\times 10^{-9}$ Pa.m.s, and $2.5\times 10^{-8}$ Pa.m.s, respectively. Given the large number of experimental pulses recovered, a median experimental pulse was computed to highlight the general trend, and shown in Figure 11a–d as a black curve.

The simulations closely reproduce the experimental signatures, particularly for the subset of results presented in Figure 11c, in which $\eta_{\text{memb}}=5\times 10^{-9}$ Pa.m.s. Figure 11b–d show that increasing $\eta_{\text{memb}}$ leads to a broader range of simulated pulse shapes in the rotation peak region. This observation is consistent with the findings reported in Section 3.2. Note also that the experimental pulses appear smoother, with a steady rise toward the peak, while simulated pulses present an initial secondary peak just before the main rotation peak. Possible explanations are discussed in Section 4.

The amplitude range of experimental pulses at t=0 is represented by a vertical black segment in Figure 11a–d. This segment indicates the rotation peak amplitude between the 5th and 95th percentiles of the experimental data. When membrane viscosity is neglected ($\eta_{\text{memb}}=0$), the simulated pulses do not fully span the range defined by this segment. Conversely, when $\eta_{\text{memb}}=2.5\times 10^{-8}$ Pa.m.s, almost 50% of the simulated pulses fall outside this range. Among the cases tested, $\eta_{\text{memb}}=5\times 10^{-9}$ Pa.m.s provides the best agreement with the experimental data, suggesting it is the most physiologically consistent value for healthy RBCs, in this configuration.

## 4 Discussion

This study presents a numerical approach for simulating Coulter‐based systems, capable of predicting both red blood cell (RBC) dynamics and the associated electric resistance signals. The numerical framework is built upon the method of Taraconat et al. [19], solving first the fluid–structure interaction problem (without RBC damage) and subsequently the electric problem, while neglecting coupling effects of the electric field on cell deformation. Regarding the numerical efficiency, the proposed framework incorporates a dynamic mesh adaptation (MA) strategy that enhances simulation flexibility while significantly reducing computational costs. Compared to the original method using a static fluid grid [19], the implementation of MA reduces the computational cost of the FSI calculation in the orifice region, resulting in an overall cost reduction of approximately 50% per pulse. Although the computational gain is already substantial, further improvements may be possible by fine‐tuning the shape of the refined region. For example, employing an ellipsoidal configuration can minimize the radial extent of the fine mesh region. In any case, the reduction in computational cost enables the execution of an extensive simulation campaign involving the systematic variation of RBC properties across a five‐dimensional parameter space. As a guideline, the simulation plan required approximately 140,000 CPU hours, corresponding roughly to 20 nodes with 128 cores operating over 2 days. While the computational cost is non‐negligible, it remains well within reach using high‐performance computing resources.

The high CPU cost of the simulations can be explained by the number of simulations to perform, but also the extreme conditions involved, including high extensional and shear stresses, high Reynolds numbers (a few hundreds in the orifice), as well as by the separation of time scales in the problem. For instance, the extension of the particles prior to their passage through the orifice is simulated over a duration of half a second while the time step is 6 orders of magnitude smaller. The YALESB2IO code uses an explicit time advancement scheme, which is constrained by time‐step stability and accuracy requirements. Although alternative methods could potentially be faster, the lack of similar applications in the literature makes it difficult to predict their possible efficiency.

The pulses exhibit a plateau, used here for non‐dimensionalizing the signals, corresponding to a period during which the RBCs flow steadily with minimal changes in deformation and orientation. This plateau is typically preceded by a rotation‐peak. The amplitude difference between the peak and the plateau serves as an indicator of the RBC shape anisotropy during rotation. This difference is sensitive to the mechanical properties of the RBC and can vary significantly from one pulse to another.

A total of five RBC parameters were investigated numerically: membrane shear modulus ($G_{s}$), cytoplasm viscosity ($\nu_{\text{in}}$), RBC volume ratio (Q), membrane viscosity ($\eta_{\text{memb}}$), and initial RBC orientation (ϕ). Except for $G_{s}$, pulse sensitivity was observed for each of these parameters across ranges corresponding to healthy RBCs. To the best of our knowledge, the effects of RBC membrane viscosity ($\eta_{\text{memb}}$) and initial orientation (ϕ) have not been previously investigated in this context. Our results show that both parameters significantly influence the pulse characteristics.

The loading time experienced by the RBCs in the orifice is short enough to allow variations in membrane viscosity and cytoplasmic viscosity to influence the RBC dynamics, even for $\eta_{\text{memb}}$ values lower than those reported in the literature for healthy RBCs. When RBC viscosity increases, the amplitude of the rotation‐peak tends to rise. This effect may be attributed to changes in the shape factor induced by increased viscosity: higher viscosity results in reduced RBC deformability. Conversely, increasing RBC sphericity leads to a reduction in the observed rotation peak, as more spherical particles exhibit less visible rotation. Consequently, a more attenuated rotation peak is expected with increasing sphericity (as reflected in Q). These tendencies corroborate previous experimental results [25] which showed that rotation peak amplitude increases when the RBC is stiffened, and decreases when the RBC is spherized.

Regarding RBC initial orientation, ϕ effects are only detected for the RBCs with the highest rigidity level. Upstream of the orifice, the extensional flow tends to stretch deformable RBCs into an elongated shape aligned with the flow direction. These shapes are approximately axisymmetric around the flow direction, so ϕ, which measures the orientation around this axis, naturally has no significant effect on the resulting dynamics and the associated pulse. However, more rigid RBCs (e.g., for $\nu_{\text{in}}=15\times 10^{-6}$ m^2^.s^−1^ or $\eta_{\text{memb}}=2.5\times 10-8$ Pa.m.s) retain a shape closer to their equilibrium discocyte shape. Moreover, the upstream flow does not alter the initial RBC orientation ϕ (c.f. Section 2.1.1), so this initial orientation is preserved. In such cases, ϕ has a significant effect, as it determines how the cell's geometry is aligned with respect to the shear stress encountered in the orifice. As suggested by Grover et al. [50], a rigid oblate particle entering the boundary layer of the orifice with an orientation $\phi =\frac{\pi }{2}$, undergoes a rolling motion around its minor axis and maintains a constant shape factor. In contrast, an initial orientation ϕ=0 leads to tumbling motion, resulting in a time‐varying shape factor. As a result, the influence of ϕ is expected to increase with RBC stiffness, with the rotation peak showing a decreasing trend as ϕ shifts from 0 to $\frac{\pi }{2}$. It is important to emphasize that the effects of ϕ and RBC rigidity, as discussed above, are significantly influenced by RBC morphology and may be different in patho‐physiological condition. For example, in spherocytosis—where RBCs become more spherical—the effects of ϕ may vanish, even at high rigidity levels. In sickle cell disease—where cells adopt a characteristic sickle shape—the effects of ϕ may become apparent even at lower rigidity thresholds.

Thereafter, all results from the systematic numerical parametric study were used to evaluate the model's capacity to reproduce the diversity of pulses observed in experiments for RBCs traveling near the orifice wall. The numerically generated pulses were qualitatively compared with experimental data acquired from a healthy blood sample analyzed using the ABX Micros 60 analyzer.

The overall shape of the pulses predicted by the simulation tool aligns well with the experimental measurements. However, systematic discrepancies are observed at the beginning of the pulses, where the simulations exhibit a secondary peak preceding the rotation peak. This effect is also observed, albeit less pronounced, at the end of the pulse plateau. Several hypotheses can be made to explain this discrepancy. First, the impact of the electric field on the RBC dynamics was not considered. However it is known that electric effects may modulate the RBC shape within the observed electric field range imposed [57, 58]. Accounting for the electric effects [59] should further improve the quality of the predictions. Second, the electric field predicted in the simulations may differ from that in the experiment, particularly near the orifice corners. In the simulations, these corners are modeled as sharp angles, whereas in reality, fabrication imperfections prevent such ideal geometries. As a result, the electric field may be overestimated at the entrance and exit of the orifice in the numerical model, which is indeed consistent with the secondary peaks present in the simulation pulses. Finally, it should be noted that the raw experimental signals are subjected to filtering to reduce measurement noise (see Section 2.2.2), which is expected to further smooth the signals and magnify the difference with simulated pulses.

Subsequently, we assessed the experimental variability in rotation‐peak amplitude and aligned it with simulation results to empirically estimate membrane viscosity. In simulations that neglect membrane viscosity, the observed experimental variation in the rotation‐peak is not fully captured. This indicates that membrane viscosity plays a critical role. Considering that reported values of RBC membrane viscosity in the literature span several orders of magnitude, various viscosity values were tested in the simulations and compared against experimental data. In the specific modeling framework used in this paper, the membrane viscosity that yields the closest agreement between the simulated and experimental pulses is $\eta_{\text{memb}}=5\times 10^{-9}$ Pa.m.s, which is an order of magnitude lower than typical values reported in the literature (see Table 1). As only a finite number of values were tested, this value should just be considered as an order of magnitude for effective membrane viscosity in the simulated regime.

To the best of our knowledge, this is the first time that RBC membrane viscosity values have been estimated in conditions where RBCs are subjected to strain rates (norm of the strain‐rate tensor) on the order of $10^{5}$–$10^{6}$ s^−1^, which are found of the ABX Micros 60 analyzer. Most measurements used to assess $\eta_{\text{memb}}$ have been conducted at significantly lower shear rates. For example, the shear rates applied in the tank‐treading experiments by Tran‐Son‐Tay et al. [30] are on the order of 1−100 s^−1^. While unmodeled phenomena—electric field effects—may influence the predictions, the discrepancy between our estimated viscosity values and those commonly reported in the literature may also be due to a shear‐thinning behavior of the RBC membrane, also reported in other studies [30, 31].

Although further efforts are needed to calibrate $\eta_{\text{memb}}$, our study demonstrates that RBCs sphericity, membrane viscosity, cytoplasmic viscosity, and its initial orientation when entering the orifice, play a key role in capturing the wide range of pulse shapes observed in the experiments. In contrast, in the range considered (from 2.2 to 22.5 μN.m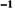) the membrane shear modulus plays a secondary role in this configuration. However, some pathological conditions can result in significantly higher values. For instance, in some cases of aging, sickle cell disease, ovalocytosis, or malaria, values of $G_{s}$ as high as 60 μN.m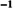 have been reported [60, 61, 62]. In such cases, the RBC shear modulus may have a measurable impact on the pulses.

This study did not explore the full parameter space of the model. Parameters that had only a minor influence on the electric signal in preliminary tests, such as the bending modulus, area dilatation modulus, and spontaneous curvature, were held constant. While these parameters may affect RBC deformation dynamics, our focus was on the electric signal, which is the sole experimental observable in this work. In particular, all simulations assumed RBCs with zero spontaneous curvature. Future work could extend the analysis by introducing non‐zero spontaneous curvature in the Helfrich bending energy or adopting more sophisticated energy formulations [51], enabling the study of alternative RBC shapes, such as stomatocytes or echinocytes, and their impact on the electric signal.

An important limitation of the model itself is the potential for RBC damage. It is well established that RBCs tolerate high stresses without damage only when exposure times are sufficiently short. Classical studies report hemolysis under prolonged exposure to high shear stresses [33], while more recent works have demonstrated sub‐lethal alterations in deformability at lower shear levels and/or shorter exposures [34]. In our configuration, RBCs locally experience high stresses (≈100–200 Pa), but only for very brief transit times (on the order of tens of microseconds). To the best of our knowledge, this short‐duration/high‐stress regime has not been characterized in the literature with respect to blood damage. As a result, sub‐lethal mechanical effects cannot be excluded. In our experiments, destruction of the RBCs would manifest as detectable changes in the electrical signal, which have not been observed. However, more subtle alterations (e.g., decreased deformability or membrane changes) may occur and could bias the inference of RBC properties, as damage‐induced changes are not accounted for in the current model. Future work should address this limitation by explicitly incorporating RBC damage into the inference framework or by exploring lower‐stress operating regimes. In particular, comparing inferred RBC parameters across varying throughputs (and thus stresses) would help identify stress thresholds beyond which mechanical damage begins to bias inferred RBC properties.

## 5 Conclusions

In this paper, we propose a numerical approach to investigate the effects of RBC properties on the impedance pulses generated in a Coulter counter (CC) during blood analysis, with the long‐term aim of extending its diagnostic capabilities to the morphological and rheological assessment of RBCs. Compared to existing numerical methods, the computational gains enabled by the MA algorithm introduced here allow us to significantly increase the number of operating points considered. This enables a sensitivity analysis of impedance pulses with respect to a broad set of RBC parameters, including factors not previously considered in this context, such as membrane viscosity.

Our numerical study, focused on healthy blood samples, confirms the influence of both morphological and rheological RBC properties on pulse shape. By comparing our results with experimental data, we identify cell sphericity, membrane viscosity, and cytoplasmic viscosity as primary contributors to the observed variability in the pulse shapes predicted by the model. In addition, we discovered that for rigid RBCs, their orientation relative to the flow direction plays a significant role—a finding not previously reported.

Nevertheless, only three discrete levels were tested for each parameter, and interactions between parameters were not fully investigated. Future work will include a more comprehensive sensitivity analysis to fully evaluate the combined effects of RBC properties on pulses. Furthermore, we will extend this study by including multiple RBC trajectories around the single one considered in this study. This generalization is expected to offer a more robust and insightful evaluation of the influence of RBC parameters on CC measurements.

Finally, to improve the inference of RBC properties from experimental measurements, future work may require refinements to the model or experimental setup to better align with real‐world conditions.

## Author Contributions

Conceptualization: Pierre Pottier, Pierre Taraconat, Damien Isèbe, Franck Nicoud, Simon Mendez. Formal analysis: Pierre Pottier. Investigation: Pierre Pottier, Pierre Taraconat, Jean‐Philippe Gineys. Methodology: Pierre Pottier, Pierre Taraconat, Damien Isèbe, Franck Nicoud, Simon Mendez. Resources: Pierre Taraconat, Jean‐Philippe Gineys, Simon Mendez. Software: Pierre Pottier, Franck Nicoud, Simon Mendez. Writing – original draft preparation: Pierre Pottier. Writing – review and Editing: Pierre Taraconat, Jean‐Philippe Gineys, Damien Isèbe, Franck Nicoud, Simon Mendez.

## Funding

This work was supported by access to high‐performance computing (HPC) resources provided by GENCI—TGCC under grants no. A0140307194 to A0180307194, and by computational resources from the Institut de Sciences des Données de Montpellier.

## Ethics Statement

In this research, human peripheral blood samples were used. We did not obtain approval from the ethics committee because the samples were obtained under a supply agreement with CHU de Montpellier, which includes provisions for their use in research. Specifically, the samples are collected during routine care and are provided anonymously to HORIBA. Patients were informed that their blood samples might be used for research and did not object. The contract stipulates that all parties adhere to French regulations regarding sample handling, ensuring respect for human dignity, integrity, and the principle of non‐ownership of the human body.

## Conflicts of Interest

The authors declare no conflicts of interest.

## Data Availability

The data that support the findings of this study are available on request from the corresponding author. The data are not publicly available due to privacy or ethical restrictions.
